# Assessing the reproducibility and up-scaling of the synthesis of Er,Yb-doped NaYF_4_-based upconverting nanoparticles and control of size, morphology, and optical properties

**DOI:** 10.1038/s41598-023-28875-8

**Published:** 2023-02-09

**Authors:** Elina Andresen, Fahima Islam, Carsten Prinz, Philipp Gehrmann, Kai Licha, Janina Roik, Sebastian Recknagel, Ute Resch-Genger

**Affiliations:** 1 Division Biophotonics, Federal Institute of Materials Research and Testing (BAM), Richard-Willstaetter-Str. 11, 12489 Berlin, Germany; 2 Division Structure Analytics, Federal Institute of Materials Research and Testing (BAM), Richard-Willstätter-Str. 11, 12489 Berlin, Germany; 3 FEW Chemicals GmbH, Technikumstraße 1, 06766 Bitterfeld-Wolfen, Germany; 4 Division Inorganic Reference Materials, Federal Institute of Materials Research and Testing (BAM), Richard-Willstaetter-Str. 11, 12489 Berlin, Germany

**Keywords:** Inorganic chemistry, Physical chemistry, Surface chemistry, Chemical synthesis, Chemistry, Nanoscience and technology, Nanoscale materials

## Abstract

Lanthanide-based, spectrally shifting, and multi-color luminescent upconverting nanoparticles (UCNPs) have received much attention in the last decades because of their applicability as reporter for bioimaging, super-resolution microscopy, and sensing as well as barcoding and anti-counterfeiting tags. A prerequisite for the broad application of UCNPs in areas such as sensing and encoding are simple, robust, and easily upscalable synthesis protocols that yield large quantities of UCNPs with sizes of 20 nm or more with precisely controlled and tunable physicochemical properties from low-cost reagents with a high reproducibility. In this context, we studied the reproducibility, robustness, and upscalability of the synthesis of β-NaYF_4_:Yb, Er UCNPs via thermal decomposition. Reaction parameters included solvent, precursor chemical compositions, ratio, and concentration. The resulting UCNPs were then examined regarding their application-relevant physicochemical properties such as size, size distribution, morphology, crystal phase, chemical composition, and photoluminescence. Based on these screening studies, we propose a small volume and high-concentration synthesis approach that can provide UCNPs with different, yet controlled size, an excellent phase purity and tunable morphology in batch sizes of up to at least 5 g which are well suited for the fabrication of sensors, printable barcodes or authentication and recycling tags.

## Introduction

Spectrally shifting upconverting nanoparticles (UCNPs), that can convert near infrared (NIR) light to luminescence photons of higher energy via a non-linear optical process, show a multitude of characteristic emission bands in the ultraviolet (UV), visible (vis), and NIR and long luminescence lifetimes, that are ideal for low background optical measurements and a high penetration depth in biological systems^1–3^. Moreover, the remarkable tunability of the upconversion luminescence (UCL) through variations of the host lattice, crystal phase, type(s) and concentrations of the dopant rare earth (RE^3+^) ions, particle size, and morphology as well as excitation conditions, i.e., excitation wavelength and power density, can be exploited for spectroscopic fingerprints in the color and lifetime domain^4,5^. This has meanwhile triggered their use as optical reporters for imaging and sensing applications^6–8^ and tags for anticounterfeiting, security, recycling, and food quality control applications^9,10^. The most frequently used crystalline host matrices for UCL-emissive UCNPs are fluorides such as NaYF_4_ because of their high transparency, very low phonon energies, and high chemical stability^11^. Doping is most frequently done with the sensitizer/activator pairs Yb^3+^/Er^3+^ and Yb^3+^/Tm^3+^, providing efficient green, red, and blue emissive UC materials. Although meanwhile many synthetic concepts for sophisticated core/multi-shell UCNPs of different size with optimized luminescence properties such as a high UCL quantum yield have been reported^12^, for many sensing, barcoding and tagging applications, simple core-only particle architectures with sizes of 25 nm or larger are completely sufficient. These UCNPs are more easily synthetically accessible and the commercial availability of such UCNPs for a reasonable price could broaden the utilization of the upconversion technology. This calls for simple and up-scalable synthesis methods for UCNPs utilizing relatively harmless and relatively inexpensive precursors that enable the controlled tuning of the UCNP physicochemical properties such as size, shape, and luminescence color.

For the synthesis of UCNPs of different size, morphology, and particle architecture, different methods have been meanwhile developed such as co-precipitation^13–15^, hydro(solvo)thermal^16–21^, thermal decomposition, and microwave assisted methods^22–24^. Up to now, the most facile method for the preparation of monodisperse UCNPs with controlled size and morphology is thermal decomposition. Thereby, the rare earth (RE) precursors are heated in a high-boiling solvent mixture in the presence of precursors of the host material. Particle growth is usually controlled by a capping ligand that stabilizes the growing nanoparticles in solution. For this purpose, commonly, oleic acid is used in conjunction with either oleylamine or trioctylphosphine^25,26^. In early reports on UCNP synthesis, mainly trifluoroacetates such as CF_3_COONa and RE(CF_3_COO)_3_ (RE = Y, Yb, Tm, Ho, and Er) were employed as RE precursors^27–30^. By careful control of parameters such as reaction time and ratio of sodium to RE trifluoroacetates, UCNP morphology could be tuned from nanospheres to hexagonal nanoplates and nanorods to nanoprisms^30^. As the pyrolysis of RE trifluoroacetates can yield highly toxic fluorinated and oxyfluorinated carbon species, later precursors such as RE acetates, prepared from RE oxides and converted to RE oleates, in combination with NaF^31^ or NH_4_F/NaOH^32^ were utilized for the synthesis of UCNPs of varying size and morphology by adjusting the ratio of oleate (OA) to octadecene (ODE) and NH_4_F or NaF. For example, in 2008, Li et al*.* reported the synthesis of a series of monodisperse β -NaYF_4_:Yb, Er, and β-NaYF_4_:Yb, Er UCNPs from RECl_3_, NH_4_F, and NaOH^33^. Na et al*.* could realize the control of the morphology of β-NaYF_4_:Yb,Er/Tm UCNPs by using a surfactant, an additive, and RE doping^34^. In this approach, the RE oleates were generated from RECl_3_ and isolated prior to their utilization for UCNP synthesis. This ensures the absence of chloride impurities and provides a better precursor solubility. The currently more common approach is, however, the in-situ preparation of RE oleates from RE chlorides and their subsequent decomposition in the presence of NH_4_F and NaOH^35,36^.

Control of the application-relevant physicochemical properties of UCNPs, that also determine their luminescence color, intensity, and brightness, requires careful control of all synthesis parameters and an in depth-understanding of the most relevant factors governing UCNP quality. This is particularly important for the up-scaling of UCNP synthesis. Synthesis parameters that can influence nanocrystal size, morphology, and crystal phase include temperature, pressure, capping ligand, precursor composition, heating rate, cooling rate, reaction time, solvent(s), and reagent concentrations. In addition, the complex mass transport dynamics associated with seed formation and nanocrystal growth require the consideration of the reaction temperature and reaction time as well as stirring speed and stirring period of the reaction mixture. This large number of synthesis variables makes the reproducible fabrication of UCNPs with specific features challenging and complicates the up-scaling of batch sizes. Furthermore, although many examples for the synthesis of NaYF_4_:Yb^3+^,Er^3+^ and the influence of different reaction parameters on the morphology of the resulting UCNPs can be found in the literature, a comparison between different studies is difficult as often different procedures including different precursors, precursor ratios, solvent ratios etc. have been employed. This was only recently demonstrated by Jurga et al*.* who examined the influence of the synthesis route on the spectroscopic and temperature-sensing properties of oleate-capped and ligand-free core/shell UCNPs produced from RE chlorides, acetates, and oleates as well as their cytotoxicity^37^.

Despite the increasing number of reports on synthesis protocols for different types of functional nanomaterials, there are still comparatively few publications tackling the reproducible synthesis of larger quantities of these materials and the up-scaling of synthesis protocols^38–43^. Common approaches for the up-scaling of nanoparticle synthesis include batch and continuous processes^44^, involving flow processes in reactors, that facilitate large-scale production via long operation times^45,46^, and microfluidic synthesis routes, as reported, e.g., for iron oxide nanoparticles^47^ and semiconductor quantum dots^48^. For UCNP synthesis, however, flow reactors or microfluidics have been scarcely used^49,50^ due to the required high nucleation temperature of > 300 °C. Until now, conventional batch synthesis remains the main synthetic procedure for large-scale UCNP production. For example, with a batch approach relying on thermal decomposition, Wilhelm et al*.* managed the first large-scale synthesis of hexagonal-phase UCNPs, that provided up to 2 g of NaYF_4_:Yb,Er nanocrystals^35^. Zhang et al*.* described a high throughput method to synthesize NaYF_4_ nanocrystals in one vessel by using liquid RE-OA precursors and increased the reaction volume with prolongated reaction times^43^, yielding about 10 g of high-quality UCNPs. You et al*.* utilized a solid–liquid thermal decomposition (SLTD) method for the fabrication of up to 63 g of β-NaGdF_4_:Yb and Er@NaYF_4_ nanoparticles in a single batch, yet employed harmful NaHF_2_ powder^51^. This encouraged us to systematically explore the influence of solvent ratio, dopant concentration, and high precursor concentrations near the solubility limit on the size, morphology, and luminescence properties of β-NaYF_4_:Yb,Er UCNPs and assess the reproducibility, robustness, and scalability of these syntheses. The overall goal of this study is to provide the basis for the reproducible and low-cost fabrication of large quantities of monodisperse simple UCNPs on a gram scale utilizing a batch synthesis approach and relatively harmless and inexpensive reagents, thereby paving the road to push the widespread use of these fascinating luminescent nanomaterials.

## Results and discussion

**Figure 1:**
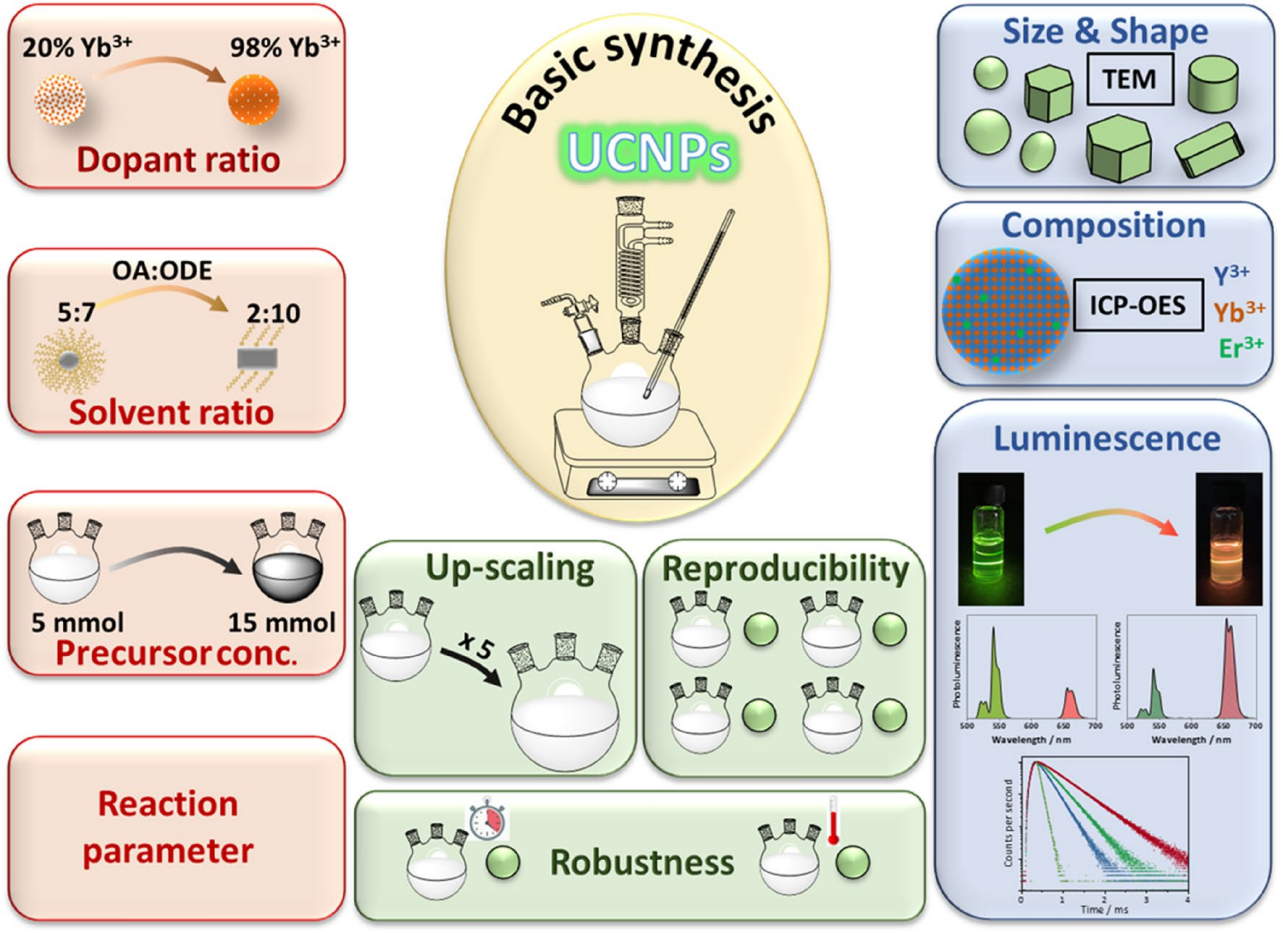
Schematic overview of the screening parameters (red, left) and the physio-chemical properties of the resulting UCNPs (blue, right) assessed for the basic synthetic approach with respect to reproducibility, robustness, and up-scaling potential (green, middle); *OA*, oleic acid, *ODE* 1-octadecene, *Robustness* reproducibility of UCNP properties as a function of or response to changes in reaction parameters such as temperature and reaction time.

Aiming for the transfer of the hot temperature UCNP synthesis from the laboratory scale of typically 100–250 mg to the reproducible production of larger quantities of few grams, we focused on a simple protocol for the synthesis of high-quality core-only UCNPs with size, morphology, and crystal phase control utilizing a batch approach. As illustrated in Fig. 1 and addressed in the following sections, this protocol can be further developed to enable the tuning of UCNP morphology and luminescence properties. This is of interest for the applications of UCNPs as fluorescent reporters in sensing schemes and as barcodes and authentication tags that require particles with different optical properties.

**Figure 2:**
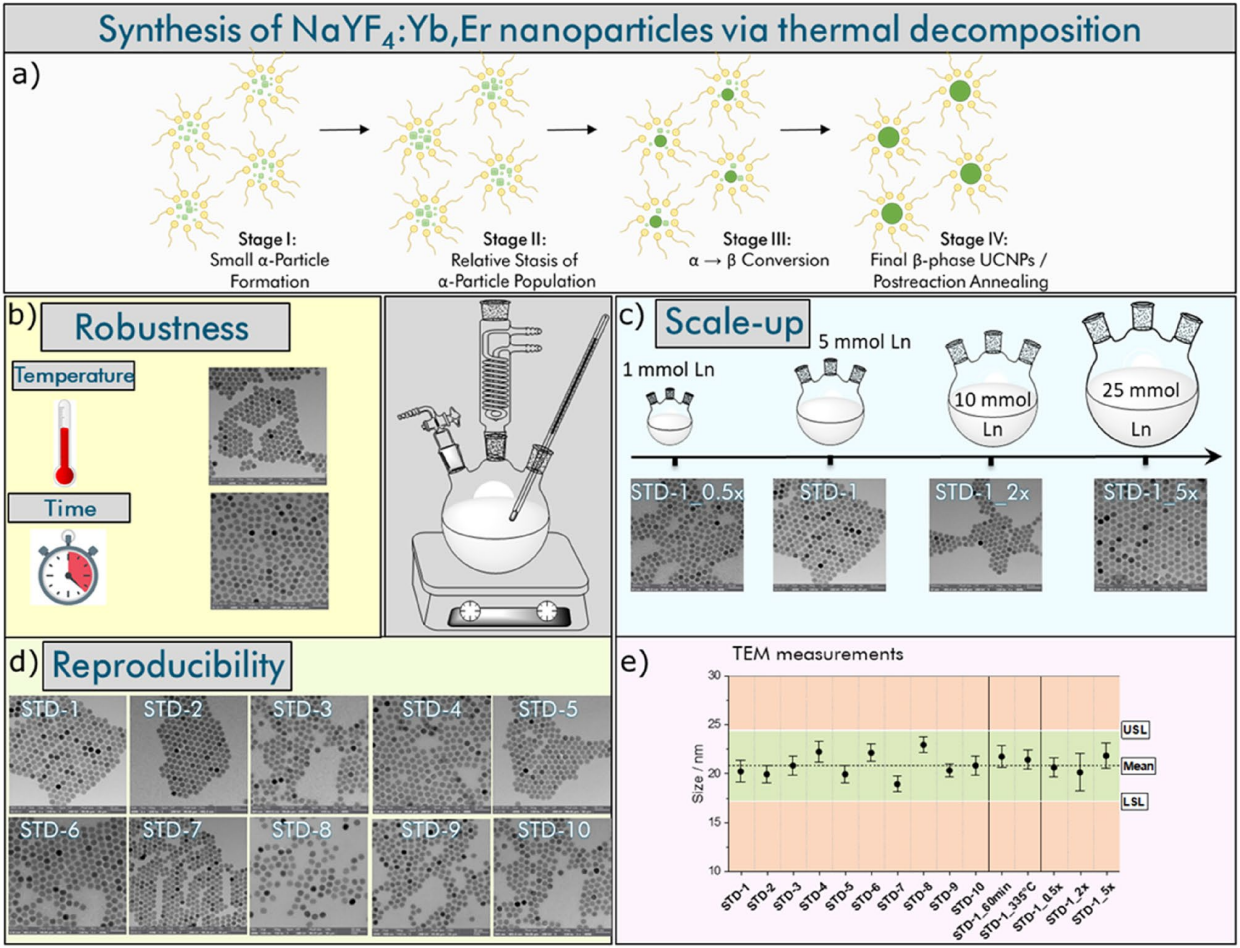
(**a**) Schematic illustration of the formation and growth of UCNPs by the thermal decomposition method according to Refs.^52,53^. The Ln precursor thermally decompose at a higher temperature to initially yield small cubic α-phase particles, which are then transformed into larger thermodynamically favored hexagonal β-phase particles via a thermodynamically driven dissolution–recrystallization process. (**b**–**e**) Reproducibility, robustness, and up-scalability of the basic synthetic procedure that yields 20 nm β-Yb,Er-co-doped NaYF_4_ UCNPs. TEM images of the UCNPs obtained from (**b**) a reaction performed with an increased temperature (335 °C instead of 325 °C) and a prolongated reaction time (60 min instead of 30 min); (**c**) different batch sizes (1–25 mmol RE precursors) to assess the robustness of the synthesis, and (**d**) ten independent reactions performed under the same reaction condition (STD 1–STD 10) to explore the reproducibility of the synthesis. (**e**) Size distribution of the obtained UCNPs as determined by TEM. TEM images of the UCNPs obtained with a higher resolution are given in the Supporting Information (SI) in Fig. S1a–o.

A prerequisite for the reproducible synthesis of nanoparticles like UCNPs is the minimization of batch-to-batch nanoparticle heterogeneity caused by variations in relevant reaction parameters. Therefore, we first examined the reproducibility, robustness, and scale-up potential of our simple synthesis protocol yielding approximately 20 nm-sized UCNPs. Subsequently, the influence of other parameters such as solvent composition, dopant ratio, and precursor concentration on the size, morphology, and luminescence properties of the obtained UCNPs was examined in screening studies detailed in the forthcoming sections. To explain the choice of the synthesis parameters addressed in this screening study, the synthesis of NaYF_4_-based UCNPs via thermal decomposition is schematically illustrated in the Fig. 2a. The formation of the UCNPs can be described by the classical La Mer model, which can be divided into three phases. In the first phase, the concentration of the precursors starts to exceed the precursor solubility in the chosen solvent. In the second phase, the concentration of the precursors reaches a supersaturation point, which then triggers nucleation and seed formation in solution. The nucleation process is associated with a decrease in the reactant/precursor concentration as some material is consumed during seed nuclei formation. In the third phase, seed growth slowly occurs. This step proceeds continuously until the reactant materials are completely depleted. For the synthesis of NaYF_4_-based UCNPs, the following reaction steps were reported for example by May et al.^52^ and Radunz et al.^53^: (i) formation of cubic phase particles (α-seeds) at temperatures below 300 °C and (ii) dissolution of the cubic particles and conversion to the thermodynamically more stable hexagonal β-phase particles (within 10 ± 2 min)^52^ following Ostwald ripening.

### Basic synthetic approach

#### Reproducibility

As an anchor point for our reproducibility, robustness, and up-scaling studies, we chose the thermal decomposition of RE precursors in high-boiling organic solvents that is well-established for the synthesis of monodisperse Yb,Er-co-doped NaYF_4_ nanocrystals. Therefore, we used the following reaction conditions: 5 mmol RECl_3_×H_2_O/12.5 mol NaOH/20 mol NH_4_F (equaling a precursor ratio of RE:Na:F = 1:2.5:4) in a mixture of 40 mL OA and 80 mL ODE, a reaction temperature of 325 °C, and a heating time of 30 min. The single steps were tightly controlled regarding duration, heating rate, and argon flow rate as described in the “Experimental section”. These standard conditions should yield spherical UCNPs with a size of approximately 20 nm. To determine the batch-to-batch reproducibility, we performed ten independent reactions (**STD-1 to STD-10**) and assessed the size and shape of the resulting UCNPs by TEM (Fig. 2d). The average size of the ten replicate reaction products is given in Fig. 2e. The error bars represent the standard deviation of UCNP size for each batch as determined by TEM. In agreement with established Statistical Process Control (SPC) methodologies, lower and upper specification limits (i.e., LSL and USL) were set to mean ± 3·σ (σ = standard deviation). The repetition of the standard synthesis yielded UCNPs with a mean size of 20.8 ± 1.2 nm equaling a variance in size of 5.7%. This value indicates only small changes in UCNP size between the different reactions and confirms the excellent reproducibility of our basic synthesis procedure.

As another parameter for the comparability of the UCNPs produced in different reactions under otherwise identical conditions, we quantified the content of the RE ions Yb^3+^, Er^3+^, and Y^3+^ of the UCNPs of the different nanoparticle batches with inductively coupled plasma-optical emission spectroscopy (ICP-OES) and compared the found amounts with the concentration of each RE reagent concentration used for UCNP synthesis. This provides also a measure for reagent consumption. The results summarized in the SI in Table S3 confirm the excellent agreement between the targeted and realized dopant ratios and the excellent reproducibility of the desired dopant concentrations of 20% Yb^3+^ and 2% Er^3+^ for all independently performed UCNP syntheses. In parallel, we examined the reproducibility of the application relevant UCNP luminescence features. The results of the steady state and time resolved luminescence measurements performed at an excitation wavelength of 980 nm and a constant *P* are summarized in the SI in Fig. S1 and Table S4. This includes the emission spectra, that provide the relative spectral distribution of the differently colored emission bands, the calculated red-to-green (R:G) ratios, and the decay kinetics (mean decay times) of the green (540 nm) and red (654 nm) emissive Er^3+^ states populated via ETU from excited Yb^3+^. The good match of the UCL spectra as well as the R:G ratios of about 0.32 ± 0.06 (1.1% variance), and the (intensity weighted) mean lifetimes of 92 ± 5 µs (6.5% variance) and 173 ± 15 µs (12.9% variance) of the green and red emission confirm the excellent reproducibility of this application relevant UCNP performance parameter.

#### Robustness

In the next step, we assessed the robustness of our UCNP synthesis for the optimized standard precursor and solvent ratios regarding the influence of the reaction temperature and reaction time, again utilizing the size, size distribution, and morphology of the resulting UCNPs as measures. These two reaction parameters are the ones which are most frequently slightly modified by chance between different reactions, often even unnoticed. As follows from the TEM images shown in Fig. 2b, also a slight increase in reaction temperature from 325 to 335 °C and a prolongation of the reaction time from 30 to 60 min barely affected UCNP size. The well reproducible size of the resulting UCNPs of about 20 nm (see entries for **STD-1_60min** and **STD-1_335°C** in Fig. 2e) underlined the robustness of our synthesis. Additionally performed ICP-OES and spectroscopic measurements assessing the impact on UCNP elemental composition and luminescence features confirmed the results of the TEM measurements. Only for sample **STD 1_60min** slightly longer decay kinetics were obtained. This is ascribed to a slight reduction in the number of crystal defects by the increased reaction time.

#### Up-scaling

Subsequently, we systematically examined the possibilities for up-scaling of our basic synthesis protocol as the batch production of UCNPs in quantities of at least 5 g is essential to broaden their applications. This batch size is sufficient for such luminescent nanoparticles given the high sensitivity of fluorescence-based detection methods down to single particles and hence the small amount of nanomaterials required, e.g., for the production of UCNP-based sensors and security inks^54,55^. For example, for the preparation of an inner filter-based hydrogel-based sensor films enabling the detection and monitoring of pH, UCNP concentrations of 2.5%, w/w with respect to the polymer were employed. This implies that with 50 mg of UCNP of a comparable size 65 cm^2^ sensor film could be obtained^54^. For inkjet printing of UCNPs for anticounterfeit applications, UCNP concentrations of 0.3 mg/mL were sufficient for a good detectability at an excitation power density of 50 mW/mm^2^ for UCNPs of comparable optical features^55^. The up-scaling of nanoparticle synthesis is particularly challenging as the complex mass transport dynamics associated with seed formation and nanocrystal growth can change with increasing reaction volume and can subsequently considerably affect the size, size distribution, and morphology of the resulting nanocrystals. Hence, we performed the UCNP synthesis according to our optimized synthetic protocol in different batch sizes of 1, 10, and 25 mmol corresponding to half-fold, two-fold, and five-fold equivalents of the batch size used for the initially developed procedure. All the precursor and solvent amounts were accordingly adapted (see SI, Table S2). As demonstrated by the TEM images (Fig. 2c) of the resulting spherical UCNPs, that all revealed sizes of about 20 nm, the straightforward scaling-up of our basic synthesis was feasible (see Fig. 2e, samples **STD-1_0.5x, STD-1_2x** and **STD-1_5x**). This straightforward scaling-up procedure can be attributed to the high amount of oleic acid (8 mL OA (7.16 g, 25.35 mmol) per mmol Ln, which equals a molar ratio OA:Ln = 5:1). During particle growth, the oleic acid acts as a capping agent with its carboxylic acid groups being coordinatively bound to lanthanide ions at the particle surface and its long hydrocarbon chain sticking into solution preventing nanoparticle aggregation by steric repulsion. Thereby, oleic acid molecules stabilize the particles during particle growth and the transition of α- into β-phase nanoparticles, ensuring the growth of monodisperse particles. This is also favored by the high nucleation and ripening temperature as well as the optimized precursor ratios. This leads to a nearly complete consumption of the starting materials during the nucleation process and avoids further Ostwald ripening of the pure β-phase particles.

We also compared the photoluminescence spectra obtained for the up-scaling series to examine the signal intensity of each UCNP batch size for a fixed particle concentration of 1 mg/mL at excitation power of 7 W/cm^2^ (see Fig. S2d). Therefore, we calculated the percentage of the green and red emission band of the different samples relative to the corresponding emission bands of the STD sample, which were set to 100%. The spectra show a maximum reduction of the luminescence intensity of the single band of 30%. Due to the high signal intensity (× 10^7^), this deviation can be attributed to small changes in UCNP concentration and/or particle size as well as to small fluctuations in excitation power density. Such effects can be elegantly circumvented by using normalized photoluminescence spectra as utilized by us for the comparison of the UCNP samples. This provides also more application-relevant information as applications in the field of (ratiometric) sensing, security and authentication tags and anticounterfeiting exploit not relative intensities of single bands but the R:G and do not refer to the single band photoluminescence intensities.

#### Synthesis with low cost RE starting materials

For the commercial production of UCNPs, another important factor presents the costs of the starting material, particularly the RE reagents. Therefore, we also explored the influence of different RE reagents on the properties of UCNPs made according to our basic synthetic approach when replacing the commonly used high-purity Ln-chlorides with less expensive alternatives like RE salts from Xi’an function material group. Surprisingly, with these reagents, UCNP synthesis yielded a black dispersion of nanoparticles which, however, were highly monodisperse and had the desired size of around 20 nm as determined by TEM (see SI, Fig. S6a). The color is ascribed to the presence of an organic impurity in the colorless starting materials, which turned black at the high reaction temperatures used. This decomposition product adsorbed onto the surface of the UCNPs. However, this impurity apparently did not influence particle growth and morphology. To confirm this assumption, we applied a typical ligand removal procedure, i.e., an acid treatment commonly used for the transfer of hydrophobic UCNPs into water^56^. This resulted in a clear and colorless aqueous dispersion of ligand-free UCNPs. TEM measurements confirmed that no change of the particle morphology was induced during the acid treatment (see SI, Fig. S6b). Luminescence measurements with these particles in organic dispersion and in water done before and after ligand removal are shown in the SI, Fig. S6c. These data support the formation of relatively strongly luminescent UCNPs also under these conditions with these inexpensive RE reagents.

In summary, our basic synthetic procedure enabled the reproducible synthesis of up to 5 g oleate-capped UCNP in a single batch (resulting from the 25 mmol batch **STD-1_5x**) of about 20 nm sized core UCNPs with size and shape control, a narrow size distribution, and matching optical properties. Aiming for the synthesis of larger UCNPs with tunable size and morphology and then variation of the luminescence features, in the next sections, subsequently we stepwise modified this synthesis approach. Then, we analogously examined the influence of synthesis parameters solvent composition, dopant concentration, and precursor concentration on the size, morphology, and optical properties of the resulting UCNPs.

### Influence of solvent on UCNP size, morphology, and luminescence

**Figure 3:**
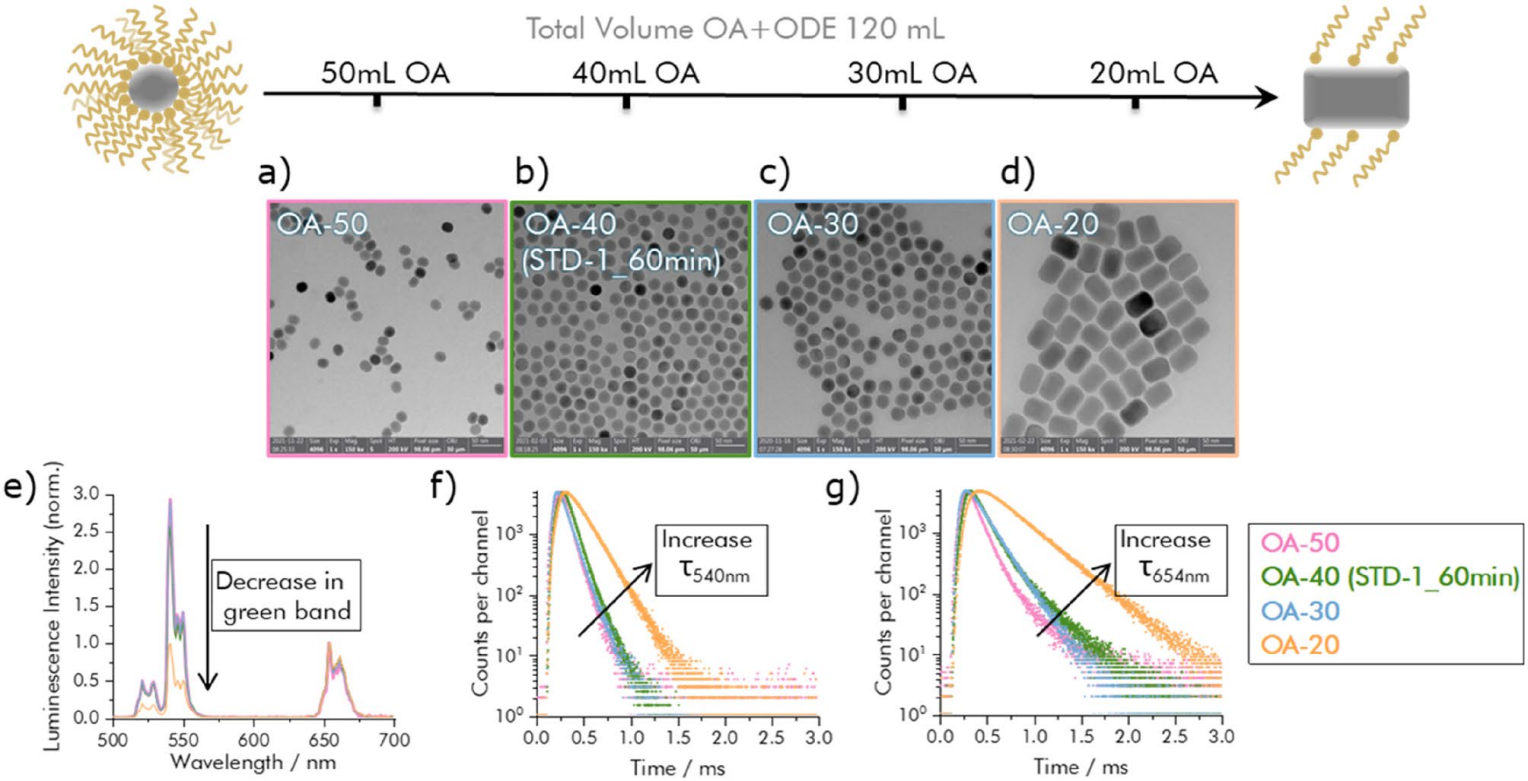
(**a**–**d**) TEM images of NaYF_4_: 2% Er^3+^, 20% Yb^3+^ synthesized using the following reaction conditions: 50 mL OA, 40 mL OA, 30 mL OA, and 20 mL OA (for TEM images with higher resolution: see SI, Fig. S1k,p–r; (**e**) UCL spectra of the resulting UCNPs, excited at 980 nm; (**f**) Decay kinetics of the 540 nm Er^3+^ emission; (**g**) Decay kinetics of the 654 nm Er^3+^ emission.

As has been shown for UCNP syntheses utilizing RE acetates^32^ or RE oleates^34^ as well as for UCNPs with a NaYbF_4_ host lattice, here by addition of the co-solvent oleylamine^26^, the variation of the solvent composition can affect the size and shape of NaYF_4_-based UCNPs. Aiming for the tuning of the application-relevant physico-chemical properties of UCNPs, we investigated the effect of solvent composition on the morphology and optical properties of UCNPs obtained by a modification of our basic synthesis approach. At first we systematically varied the ratio of the solvents OA and ODE while the amount of the different RE ions, i.e., the corresponding RE^3+^-chloride precursors, and the total solvent volume were kept constant at 5 mmol and 120 mL, respectively. All other synthesis parameters were chosen to be identical with the parameters utilized for our previously introduced basic synthesis. The amount of OA was then reduced from 50 to 20 mL in 10 mL steps. The reatcion time was set to 60 min to ensure the complete conversion of the precursor materials. Figure 3a–d shows the TEM images of the resulting UCNPS. These TEM images clearly show the size and morphology evolution of the NaYF_4_:Yb,Er UCNPs from spheres to rods accompanying these modifications of the synthesis parameters. A solvent composition of 40 mL OA and 80 mL ODE favored an isotropic particle growth, yielding monodisperse and highly uniform UCNPs with a size of approximately 21.7 nm and a spherical shape. Increasing the OA amount from 20 to 50 mL (sample **OA-50**) yielded slightly smaller particles of elliptical shape with a length (l) of 21.6 ± 1.5 nm and width (w) of 18.3 ± 1.4 nm. When the OA volume was decreased to 30 mL (sample **OA-30**), the particle size increased to 26.5 nm, yet the particle shape remained spherical. A further reduction in OA volume to 20 mL resulted in significantly larger rod-shaped UCNPs (sample **OA-20**) with l = 53.6 ± 3.2 nm and w = 38.4 ± 3.4 nm. These findings are consistent with the observations reported in previous studies by Li et al*.* and Na et al.^32,34^. We ascribe this evolution of UCNP size and shape to the interaction of the OA capping ligands with the growing nanocrystals in the non-coordinating solvent ODE. The thermal decomposition of the RE oleates at high temperature produces small nuclei in the reaction mixture. When the nuclei grow, the carboxylic acid groups of the OA molecules strongly interact with a particular crystallographic plane of the growing nanoparticles. The strength of this interaction depends on the density of the RE surface atoms of the UCNP nuclei and crystals and is particularly strong for a crystallographic plane with a high density of RE surface atoms. For a hexagonal, closely packed (HCP) crystal structure, the density of the surface atoms is higher in [001] than in [100] systems. The adsorption of capping ligands or surfactants on the [001] facets slows down the particle growth rate along this direction or even prevents UCNP growth. As a result, the UCNPs grow faster along the [100] direction compared to the growth along the [001] direction, thus leading to the formation of nanorods.

The luminescence properties of the UCNPs from this solvent series considerably differed as can be seen in the panels e–g of Fig. 3, although the material composition was identical as confirmed by ICP-OES measurements shown in the SI (Table S3). The calculated R/G ratios, that are summarized in the SI in Table S4, reveal that the green emission of sample **OA-20** is strongly reduced (R:G = 0.89) compared to sample **OA-40** (R:G = 0.32). Also, with values of 107 µs and 307 µs, the lifetimes of the 540 nm and 654 nm UCL emission bands of **OA-40** are enhanced. These changes in the luminescence features are attributed to surface effects and surface quenching by the presence of high vibrational energy groups of the ligands, e.g., –CH and –OH groups, which are more pronounced for smaller particles with a higher surface area-to-volume ratio^35,57^. Also, the possible formation of surface defects like RE vacancies and/or lanthanide segregation^57^ could contribute to luminescence quenching.

### Dopant concentration effects

**Figure 4:**
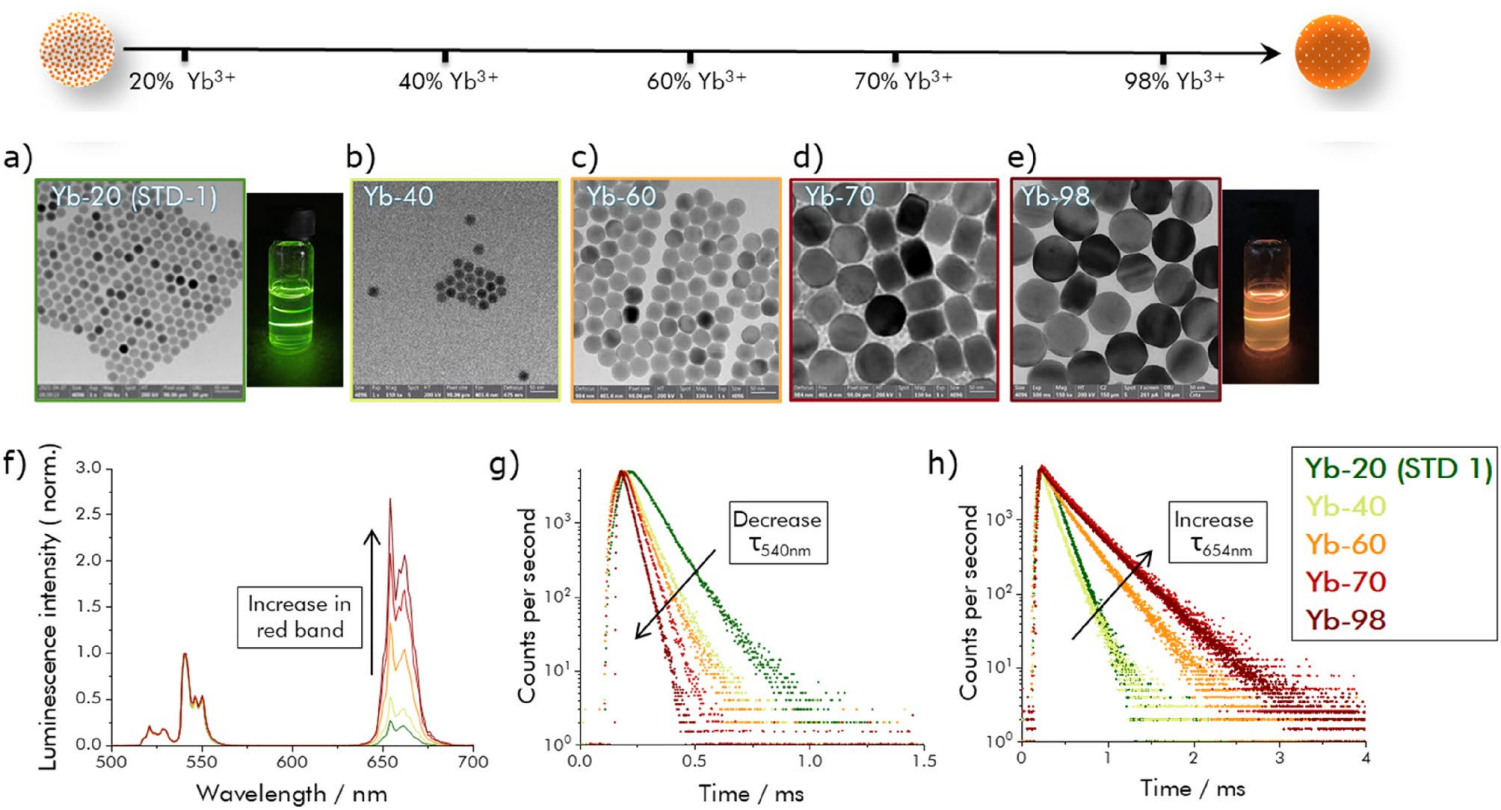
(**a**–**e**) TEM images of the NaYF_4_ UCNPs co-doped with 2% Er^3+^ and various concentrations of Yb^3+^: 20% Yb^3+^, 40% Yb^3+^, 60% Yb^3+^, 70% Yb^3+^, and NaYbF_4_: 2% Er^3+^. For TEM images with higher resolution: see SI, Fig. S1a,s–v. The photographs in the insets show the visible emission of each sample under 980 nm excitation; (**f**) UCL emission spectra of NaYF_4_: x % Yb^3+^, 2% Er^3+^ UCNPs under 980 nm excitation; (**g**) Decay kinetics of the 540 nm Er^3+^ emission; (**h**) Decay kinetics of the 654 nm Er^3+^ emission.

To tune UCL emission color, we explored the influence of the Yb^3+^ concentration on the size, morphology, and luminescence properties of core-only UCNPs. Therefore, the Yb^3+^ concentration was stepwise increased from 20 to 100% while simultaneously reducing the amount of Y^3+^. All other reaction parameters were identical with the previously optimized basic synthesis protocol. Panels a–e in Fig. 4 display the TEM images of the NaYF_4_: x % Yb^3+^, 2% Er^3+^ UCNPs prepared with x equaling 20, 40, 60, and 70% Yb^3+^ as well as pure NaYbF_4_:2% Er^3+^. Apparently, with an increase in Yb^3+^ concentration, the size and morphology of the resulting UCNPs changed from 20 nm sized spherical nanocrystals, obtained for an Yb^3+^ concentration of 20%, to 40 nm sized disc-like particles for an Yb^3+^ concentration of 60% (Fig. 4c). At higher Yb^3+^ concentrations of 70% and 98%, the nanocrystal shape remained disc-like, yet the size further increased, yielding UCNPs with a diameter (d) of 70.4 ± 1.7 nm and a width (w) of 50.1 ± 1.7 nm and d = 87.5 ± 3.7 nm and w = 66.2 ± 2.1 nm, respectively (Fig. 4d,e). The corresponding size histograms revealing the average size distribution are summarized in the SI in Fig. S1s–v. As confirmed by ICP-OES measurements, the elemental composition of all UCNPs was always in good agreement with the initial Y^3+^, Yb^3+^, and Er^3+^ precursor ratios (see SI, Table S1).

The observed Yb^3+^-induced size and shape evolution of the UCNPs, that was also reported by other groups^58^, is attributed to the Yb^3+^-controlled crystal growth rate involving the modification of the electron charge density on the nanoparticle surface. The opposite trend, i.e., a reduction in particle size by replacing Y^3+^ ions (ion radius of 0.893 Å) in the NaYF_4_ matrix by larger Gd^3+^ ions (ion radius of 0.938 Å) has been more frequently examined. Therefore, Gd^3+^ ions are not only exploited for the design of multimodale probes for theranostic applications detectable with optical and magnetic methods^59^, but also for the preparation of small and ultrasmall UCNPs with sizes below 10 nm^60^. Based on density functional theory (DFT)-calculations, the particle growth behavior in the presence of higher amounts of Gd^3+^ ions, yielding an increased electron density of the crystal surface and hence a more negative charge of the UCNPs, is ascribed to the electrostatic repulsion of the fluoride anions. This favors the formation of smaller UCNPs^61^. When Y^3+^ ions (ion radius of 0.893 Å) are substituted by smaller Yb^3+^ ions (ion radius of 0.868 Å), the electron density on the UCNP surface is reduced. This facilitates the interaction with fluoride^-^ anions and results in the growth of larger UCNPs.

Subsequently, the crystalline phases of the as-prepared UCNP samples of the Yb^3+^ dopant concentration series were examined by powder XRD. The diffractograms are shown in the SI in Fig. S2 in comparison to hexagonal phase NaYF_4_ (JCPDS 16-0334) and hexagonal phase NaYbF_4_ (JCPDS 27-1427) UCNPs. These measurements confirmed the formation of a hexagonal crystal phase for all prepared samples. With increasing Yb^3+^ concentration, the XRD peaks shift to high angles. This is indicative of a decrease of the unit cell volume caused by the replacement of Y^3+^ by the smaller Yb^3+^ ions. Apparently, the hexagonal phase NaYF_4_ crystalline host matrix is gradually transformed to a hexagonal phase NaYbF_4_ crystalline host matrix with elevated Yb^3+^ doping concentrations.

The size and morphology changes induced by increased Yb^3+^ doping concentrations were also accompanied by changes in the luminescence properties of the resulting UCNPs. As shown in Fig. 4f, an increasing Yb^3+^ concentration led to a gradual increase in the red Er^3+^ UCL emission relative to the green Er^3+^ UCL emission. For NaYbF_4_:2% Er UCNPs (**Yb-98**), the red emission was enhanced by a factor of about 15 compared to UCNPs containing the commonly used standard concentration of 20% Yb^3+^ (**Yb-20**). This is also reflected by a change in the R:G ratio from, e.g., 0.32 to 2.25 for Yb^3+^ doping concentrations of 20% and 70%, respectively, as well as by changes in the decay kinetics and luminescence lifetimes of the Er^3+^ emission that is populated by ETU from the initially excited Yb^3+^ ions. These effects are displayed in Fig. 4g,h, revealing a steady decrease of the lifetime of the green Er^3+^ emission from 93 to 33 µs and a concomitant increase in the lifetime of the red Er^3+^ emission from 152 to 366 µs for the UCNPs with the lowest (**Yb-20**) and highest (**Yb-98**) Yb^3+^ dopant concentrations used. The corresponding intensity-weighted lifetimes are provided in the SI in Table S4. The downconverted luminescence (DCL) decay of Yb^3+^ recorded at 1000 nm showed a rapid diminution in lifetime for Yb^3+^ concentrations > 20% (see SI, Fig. S4). This indicates that higher Yb^3+^ concentrations increase the probability that the excitation energy reaches quenching sites, e.g., at the particle surface due to faster energy migration. In addition, for our deliberately relatively simple synthesis, the introduction of additional quenching sites and crystal defects by an increased Yb^3+^ concentration cannot be excluded. For Yb^3+^ concentrations exceeding 25%, also back energy transfer (BET) from Er^3+^ ions to Yb^3+^ ions is principally possible, which could also favor the population of the red emissive Er^3+^ energy level ^4^F_9/2_. Similar UCL effects have been recently reported for an Yb^3+^ concentration series of UCNPs, i.e., core/shell UCNPs prepared by an elaborate water-free synthesis that yields high quality almost defect-free UCNPs which show the highest UC quantum yields reported so far for NaYF_4_:Yb, Er UCNPs doped with 20% Yb^3+^ and 2% Er^3+^^62^ and still high UC quantum yields even for high Yb^3+^ doping concentrations^63^. Although for this elaborately made UCNPs, very high Yb^3+^ doping concentrations slightly reduce the UC quantum yield, nevertheless the strongly enhanced absorption cross section of the UCNPs boosts UCNP brightness^63^.

As the relative spectral contribution of the different emission bands to overall UCL depend on *P*^64^, the emission spectra of the UCNPs from the Yb^3+^ concentration series were measured at *P* values ranging from 7 to 130 W cm^−2^. For all samples, the intensity ratio of the R:G ratio increased for increasing *P* as shown in the SI in Fig. S5. For Yb^3+^ concentrations < 40%, the relative spectral contribution of the green Er^3+^ emission decreased but nevertheless remained dominant in the studied *P* range. For the sample **Yb-40**, the intensity of the green and red Er^3+^ emission bands match, i.e., cross at *P* of 50 W cm^−2^. However, for higher Yb^3+^ doping concentrations (samples **Yb-60**, **Yb-70** and **Yb-98**), the red emission band becomes dominant even at low *P* and its relative spectral contribution is further increased for higher *P*. This favors the population of high Er^3+^ energy levels involving population processes of higher photonic order^65^.

### Precursor concentration effects

**Figure 5:**
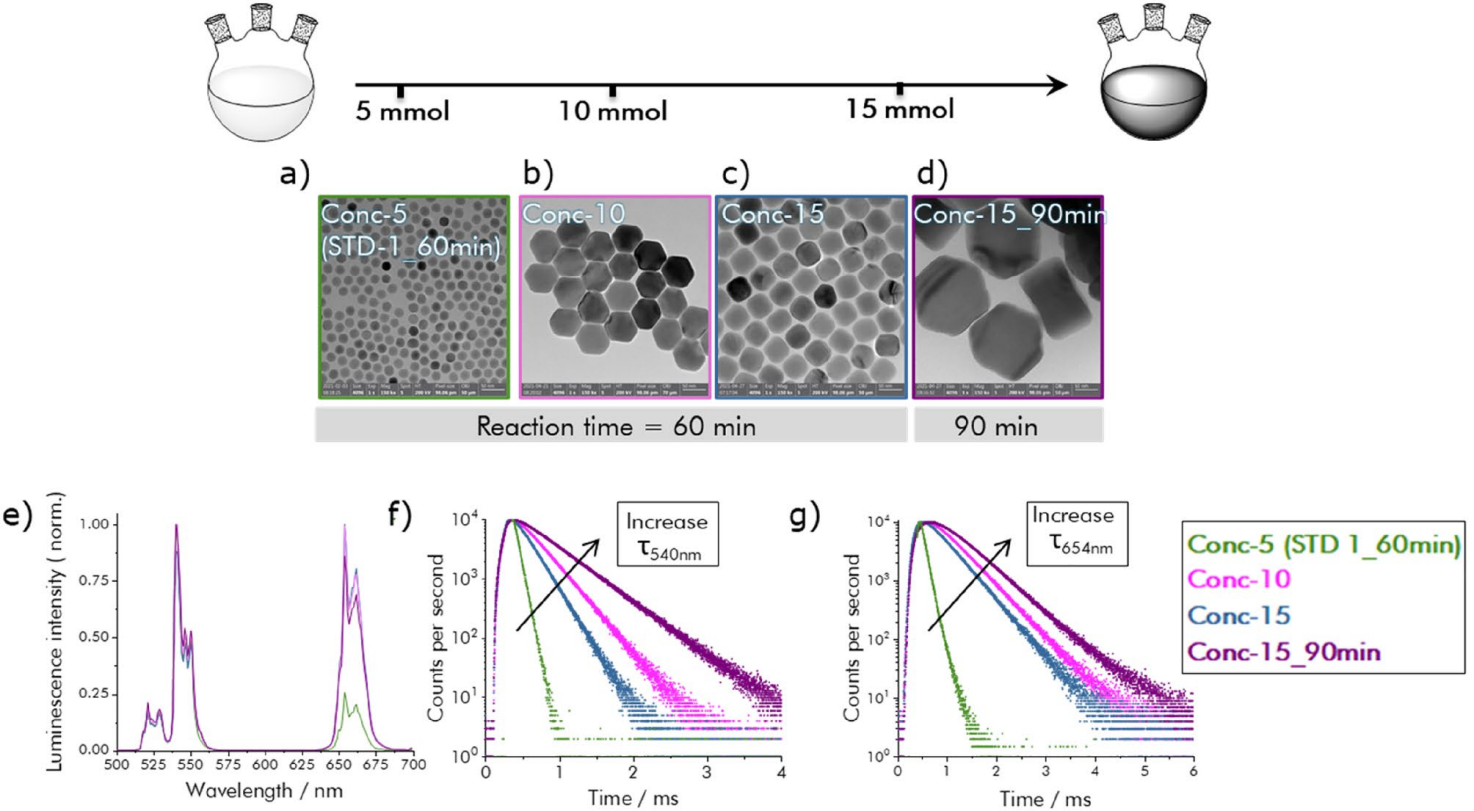
(**a**–**d**) TEM images of NaYF_4_ nanocrystals co-doped with 20% Yb^3+^ and 2% Er^3+^ synthesized by increasing the RE precurcors concentration in a constant solvent volume (120 mL): 5 mmol, 10 mmol, and 15 mmol LnCl_3_; for TEM images with higher resolution: see SI, Fig. S1k,w–y; (**e**) UCL emission spectra of NaYF_4_: x % Yb^3+^, 2% Er^3+^ UCNPs under 980 nm excitation; (**f**) Decay kinetics of the 540 nm Er^3+^ emission; (**g**) Decay kinetics of the 654 nm Er^3+^ emission.

Finally, as a high precursor conversion is important for an efficient use of the starting materials and solvent and thus also for material production costs, we performed screening experiments to assess the influence of the precursor concentration on the size, morphology, and luminescence features of the resulting UCNPs. Specifically, we examined a previously underexplored parameter of UCNP synthesis, the synthesis in the saturation regime of the RE precursors. Therefore, we increased the precursor concentrations up to threefold beyond the amount of reagents employed for our basic synthesis procedure. Three independent experiments were performed utilizing 42 mM (standard (**Conc-5** sample (**STD-1**)), 84 mM (**Conc-10** sample), and 125 mM (**Conc-15** sample) reaction mixtures under otherwise identical synthesis conditions and the obtained particles are shown in Fig. 5a–c. Duplication of the precursor concentrations led to UCNPs with a more pronounced hexagonal shape with a diameter of 71.8 ± 2.7 nm and a width of 53.2 ± 2.7 nm. Interestingly, triplication of the precursor amounts yielded 40 nm sized UCNPs of nearly spherical shape. For this reaction mixture, an exemplarily performed prolongation of the reaction time to 90 min resulted in the formation of nanoplates with a diameter of 179.2 ± 5.8 nm and a width of 101.1 ± 4.5 nm (Fig. 5d). ICP-OES measurements revealed that in all cases, the resulting UCNPs contained the desired doping concentration of 20% Yb and 2% Er, underlining efficient precursor conversion.

According to the LaMer model, under identical heating and synthetic conditions, the nucleation rate depends on the degree of (super)saturation and thus, on the initial precursor concentrations. An increase in the amount of RECl_3_/NaOH/NH_4_F in the reaction mixture favors saturation of the reaction cocktail and thus, the formation of many nuclei within a short reaction time, while a small amount of the precursor species remains in the reaction mixture promoting particle growth. This leads to smaller particles for the **Conc-15** sample compared to the **Conc-10** sample. Another effect, that can accompany increased precursor concentrations and can possibly also play a role here, is an increase in viscosity of the reaction mixture. To gain an improved understanding of the interplay between chemical, thermal, and rheological properties of the reaction mixture on nucleation and particle growth required for size and morphology control and low production costs, more detailed and systematic studies are required.

As to be expected, the differences in particle size and morphology strongly affected the optical properties of the formed UCNPs. This is highlighted in Fig. 5e–g. The UCL emission spectra of all samples exhibit a strong increase in the red Er^3+^ emission compared to the sample **STD-1_60min**. The small variations between the samples **Conc-10**, **Conc-15**, and **Conc-15_90min** suggest that for particle sizes exceeding 50 nm, the R:G ratio remains more or less constant. This is attributed to the decreased surface-to-volume ratio of larger particles, which strongly reduces the influence of surface defects and surface quenching on UCL. More pronounced differences between the different samples can be observed by time-resolved luminescence measurements shown in the SI in Table S4. As highlighted in this table, the lifetime of the green emissive Er^3+^ energy level gradually increased with increasing particle size. Also, the lifetime of the red Er^3+^ emission rapidly increased for an increase in particle size from 20 to 40 nm, followed by a smaller prolongation in lifetime for the samples **Conc-10** (60 nm) and **Conc-15_90min** (d = 179.2 ± 5.8 nm and w = 101.1 ± 4.5 nm). These effects are ascribed to the often observed increase in UCL efficiency with increasing particle size.

For the UCNP sample **Conc-10**, we representatively assessed the possibility to up-scale batch size and performed the synthesis under identical conditions on a 4-times larger scale, using RE precursor concentrations of 40 mmol and a 1L flask (**Conc-10 × 4**). The TEM images of the resulting UCNPs, shown in the SI (Fig. S1z), reveal hexagonal shaped particles with a diameter of 58.4 ± 2.3 nm and width of 39.1 ± 1.2 nm. This is in good agreement with sample **Conc-10** and demonstrates the robust nature of UCNP synthesis in the highly concentrated precursor regime. Apparently, also for these reaction conditions, up-scaling and the synthesis of larger batch sizes are feasible.

## Conclusion and outlook

Aiming for the identification of reaction conditions that are suitable for the large scale production of simple core-only β-NaYF_4_:Yb, Er upconverting nanoparticles (UCNPs) for applications such as sensing and authentication, security, and release tags, we developed a robust, reproducible, and up-scalable thermal decomposition synthesis protocol. Under optimized reaction conditions, this synthesis enables the preparation of batch sizes of 5 g of monodisperse UCNPs, here 21 nm-sized particles with a batch-to-batch variability in UCNP size of only ~ 6%. Subsequently performed screening studies to explore the parameters reaction time, temperature, and batch size did not reveal changes in particle size and morphology. Screening of the parameters dopant concentration, solvent composition, and precursor concentration showed that the particle size and morphology as well as the optical properties can be modified by varying a single synthetic parameter. Variation of the ratios of the solvents oleic acid (OA) and octadecene (ODE) led to a slight increase in particle size for OA:ODE ratios of 5:7 to 3:9, followed by a rapid increase in size and change in shape for lower OA amounts. Variations of the Yb^3+^ dopant concentration from 20 to 100% induced changes in UCNP size and, as to be expected, the strongest modulation of the optical properties. Screening studies with different rare earth (RE) precursor concentrations and highly concentrated reaction mixtures highlight the influence of this barely explored reaction parameter, that can influence the growth kinetics and thereby particle morphology and illustrates its potential for the tuning of UCNP size. ICP-OES measurements done with all UCNPs demonstrated that the RE ions Y^3+^, Yb^3+^, and Er^3+^ were always quantitatively incorporated into the resulting UCNPs. This confirmed the precise control of the dopant concentration by the choice of the RE precursor amount.

In summary, we demonstrated the robust scale-up of the synthesis of simple UCNPs by a thermal decomposition route utilizing relatively inexpensive precursors. Moreover, we could identify synthesis parameters to further tune the application-relevant features of these UCNPs. These findings will pave the road to a platform of UCNPs with sizes from 20 to 150 nm with tunable optical properties, that can be produced in large quantities at relatively low costs, most likely also in an industrial environment. In the future, we plan to systematically study our high precursor concentration synthesis procedure to gain an in-depth understanding of the interplay between chemical, thermal, and rheological properties for this yet underexplored approach. Moreover, we will also examine shorter heating rates, lower temperatures, and different reaction times for different batch sizes to further reduce costs for material production.

## Experimental section

### Chemicals

YCl_3_·6H_2_O (99.99%), YbCl_3_·6H_2_O (99.99%), ErCl_3_·6H_2_O (99.99%), oleic acid (90% technical grade), and NaOH (98%) were purchased from Sigma-Aldrich. 1-octadecene (ODE, 90% technical grade) and NH_4_F (99.99%) were obtained from Alfa Aesar. Chloroform, cyclohexane, acetone, and ethanol were purchased from Carl Roth GmbH. Low-price Ln-chlorides were puchased from XI’AN FUNCTION MATERIAL GROUP. All chemicals were used without further purification.

### General synthetic route for oleate-capped NaYF_4_:Yb^3+^,Er^3+^ (basic synthetic approach)

Co-doped oleate (OA)-capped NaYF_4_:Yb^3+^,Er^3+^ were synthesized following a procedure from Wilhelm et al*.* for the large-scale synthesis of hexagonal-phase UCNPs^35^. Briefly, YCl_3_·6H_2_O (1183.10 mg, 3.80 mmol), YbCl_3_·6H_2_O (387.50 mg, 1 mmol), and ErCl_3_·6H_2_O (38.17 mg, 0.20 mmol) were dissolved in 5 mL of methanol by sonication and subsequently added to a mixture of oleic acid (40 mL) and 1-octadecene (80 mL) in a 250 mL three-necked flask. The stirred reaction mixture was then heated to 150 °C under argon flow. After 30 min, vacuum was applied for further 30 min at 150 °C to remove remaining low-boiling impurities. The RE precursor-containing reaction mixture was then cooled down to room temperature (rt) under a constant argon flow. Subsequently, a methanolic solution (10 mL) containing NaOH (500 mg, 12.5 mmol) and NH_4_F (740 mg, 20 mmol) was added, and the resulting suspension was heated to 120 °C for 30 min to remove excess methanol. The reaction mixture was then heated to 325 °C under reflux under a gentle argon flow, kept at this temperature for 25 min., and cooled down to room temperature (rt). The resulting UCNPs were purified following a literature procedure^35^, dispersed in cyclohexane (c = 30 mg/mL), and stored at 4 °C. To study the influence of different reaction parameters on UCNP size, morphology, and optical properties, this basic synthetic approach was modified with respect to reaction time and temperature, solvent ratio, precursor ratio and concentration as well as batch size. The reaction conditions and the subsequently used nomenclature are detailed in the Supporting Information (SI) in Table S1.

### Preparation of ligand-free NaYF_4_:Yb^3+^,Er^3+^

Oleate-capped UCNPs were isolated by centrifugation (30 min, 10,000 rpm). Removal of the oleate ligands was achieved by dispersing these UCNPs in aqueous HCl (0.3 M) solution and treating them in an ultrasonic bath for 30 min at room temperature (rt). When the reaction was completed, the aqueous solution was extracted three times with diethyl ether to remove the oleate ligands. The UCNPs in the water phase were then collected by centrifugation (30 min, 10,000 rpm) and washed twice with milliQ water. Finally, the ligand free UCNPs were redispersed in water and stored at 4 °C.

### Analytical characterization and luminescence spectroscopy

Parts of the method descriptions given in the analytical characterization and steady state and time-resolved luminescence spectroscopy sections have been taken from Ref.^56^.

*Transmission electron microscopy* (TEM) images were obtained with a Talos F200S Microscope (Thermo Fisher Scientific) using an acceleration voltage of the electron beam of 200 kV. The samples were prepared by dropping UCNP dispersions (c = 1 mg/mL in water) onto a 3 mm copper grid (lacey, 400 mesh) and allowing them to dry under air at rt. The TEM images were analyzed with the software ImageJ. For as synthesized OA-capped UCNPs, the average particle size was determined from 8 micrographs (58kx magnification), each containing approximately 350 particles. Thereby, the particle area was automatically measured using a fixed threshold based on unprocessed image intensity histograms and size distribution descriptors (e.g., Feret_max_ and Feret_min_). The obtained diameters were plotted in the form of a histogram which was subsequently fitted with a Gaussian curve. The mean (µ) and standard deviation (σ_x_) of this curve were taken as representative particle size for the respective sample.

*X-ray diffraction* (XRD) measurements providing patterns that give information on UCNP crystal phase were done with a Rigaku Ultima IV diffractometer (Rigaku, Tokio, Japan) in the range of 10–80°/2θ utilizing Cu Kα radiation (λ = 0.15406 nm). The acceleration voltage was 40 kV, and the current was 40 mA. The scanning step was 0.2°/2θ with a counting time of 4 s per step.

*Inductively coupled plasma optical emission spectrometry* (ICP-OES) was applied to quantify the amount of RE^3+^ ions in the UCNPs. The ICP-OES measurements were done with a SPECTRO Arcos-EOP (Model: FHX, 76004553) spectrometer. The ICP-OES standard solutions containing 1000 mg/L of the RE^3+^ ions Yb^3+^, Er^3+^, and Y^3+^ in nitric acid (2–3%) employed for the calibration required for RE^3+^ quantification were purchased from Sigma Aldrich. The calibration was performed with 10 standard solutions covering the concentration range of 0–2000 µg/L, 0–200 µg/L and 0–7200 µg/L for Yb^3+^, Er^3+^, and Y^3+^, respectively. The UCNP samples were dried, dissolved in nitric acid, and further diluted in milliQ water prior to the ICP-OES measurements.

### Steady state and time-resolved luminescence spectroscopy

Spectrally resolved UCL measurements were carried out on an Edinburgh Instrument spectrofluorometer, model FLS980-xD2-stm, equipped with an 8 W, 978 nm laser diode. For luminescence decay measurements, an electrically pulsed, 8 W, 978 nm laser diode (long square pulses, pulse width of 150 μs) was used. The decay kinetics were recorded at 540 nm (green Er^3+^ UCL), 655 nm (red Er^3+^ UCL), and 1000 nm (down converted luminescence (DCL) of Yb^3+^) with a red-sensitive photomultiplier tube (PMT; Model H10720-20) from Hamamatsu, using time-correlated single photon counting (TCSPC). All measurements were performed at the same excitation power density (*P*). For *P*-dependent time-resolved luminescence measurements, *P* was varied (pulse width kept constant) and the emission slit width of the monochromator was modified in such a way that always the same number of photon counts per second (cps) of 2000 was detected. Luminescence lifetimes were calculated from the measured decay kinetics with the FAST software from Edinburgh Instruments using a second-order exponential decay fit. The decay curves of the long-lived UCL were used as obtained, without consideration of the instrument response function (tail fit, no unfolding of the instrument response function).

## Supplementary Information

Supplementary Information.

## Data Availability

All data generated or analyzed during this study are included in this published article (and the Supporting Information files) or are available from the corresponding authors on reasonable request.

## References

[1] Chen GY, Qju HL, Prasad PN, Chen XY (2014). Upconversion nanoparticles: Design, nanochemistry, and applications in theranostics. Chem. Rev..

[2] Haase M, Schafer H (2011). Upconverting nanoparticles. Angew. Chem. Int. Ed..

[3] Escudero A (2017). Rare earth based nanostructured materials: Synthesis, functionalization, properties and bioimaging and biosensing applications. Nanophotonics.

[4] Resch-Genger U, Gorris HH (2017). Perspectives and challenges of photon-upconversion nanoparticles—Part I: Routes to brighter particles and quantitative spectroscopic studies. Anal. Bioanal. Chem..

[5] Himmelstoss SF, Hirsch T (2019). A critical comparison of lanthanide based upconversion nanoparticles to fluorescent proteins, semiconductor quantum dots, and carbon dots for use in optical sensing and imaging. Methods Appl. Fluoresc..

[6] Brandmeier JC (2021). Effect of particle size and surface chemistry of photon-upconversion nanoparticles on analog and digital immunoassays for cardiac troponin. Adv. Healthc. Mater..

[7] Kale V (2016). Spectrally and spatially multiplexed serological array-in-well assay utilizing two-color upconversion luminescence imaging. Anal. Chem..

[8] Arai MS, de Camargo ASS (2021). Exploring the use of upconversion nanoparticles in chemical and biological sensors: From surface modifications to point-of-care devices. Nanoscale Adv..

[9] Peltomaa R, Benito-Peña E, Gorris HH, Moreno-Bondi MC (2021). Biosensing based on upconversion nanoparticles for food quality and safety applications. Analyst.

[10] Suo H (2021). High-security anti-counterfeiting through upconversion luminescence. Mater. Today Phys..

[11] Tiwari SP (2018). Future prospects of fluoride based upconversion nanoparticles for emerging applications in biomedical and energy harvesting. J. Vacuum Sci. Technol. B..

[12] Hudry D (2021). Interface pattern engineering in core-shell upconverting nanocrystals: Shedding light on critical parameters and consequences for the photoluminescence properties. Small.

[13] Lin M (2012). Recent advances in synthesis and surface modification of lanthanide-doped upconversion nanoparticles for biomedical applications. Biotechnol. Adv..

[14] Yi G (2004). Synthesis, characterization, and biological application of size-controlled nanocrystalline NaYF4:Yb,Er infrared-to-visible up-conversion phosphors. Nano Lett..

[15] Yang Z, Gredin P, Mortier M (2019). Extremely straightforward room temperature co-precipitation method to synthesize cubic KYF4:Yb/Er up-conversion nanoparticles in deionized water-ethanol solution. Opt. Mater..

[16] Rafique R (2019). A facile hydrothermal synthesis of highly luminescent NaYF4:Yb^3^^+^/Er^3^^+^ upconversion nanoparticles and their biomonitoring capability. Mater. Sci. Eng. C.

[17] Ding M (2015). Hexagonal NaYF4:Yb^3^^+^/Er^3^^+^ nano/micro-structures: Controlled hydrothermal synthesis and morphology-dependent upconversion luminescence. Appl. Surf. Sci..

[18] Li S (2017). OH− ions-controlled synthesis and upconversion luminescence properties of NaYF4:Yb^3^^+^, Er^3^^+^ nanocrystals via oleic acid-assisted hydrothermal process. J. Rare Earths.

[19] Qiu H (2011). Ethylenediaminetetraacetic acid (EDTA)-controlled synthesis of multicolor lanthanide doped BaYF5 upconversion nanocrystals. J. Mater. Chem..

[20] Lin H (2016). Morphology evolution and pure red upconversion mechanism of β-NaLuF4 crystals. Sci. Rep..

[21] Qiu P (2015). An anion-induced hydrothermal oriented-explosive strategy for the synthesis of porous upconversion nanocrystals. Theranostics.

[22] Wang H-Q, Nann T (2009). Monodisperse upconverting nanocrystals by microwave-assisted synthesis. ACS Nano.

[23] Wang H-Q, Tilley RD, Nann T (2010). Size and shape evolution of upconverting nanoparticles using microwave assisted synthesis. Cryst. Eng. Comm..

[24] Halimi I (2019). Pick your precursor! Tailoring the size and crystal phase of microwave-synthesized sub-10 nm upconverting nanoparticles. J. Mater. Chem. C.

[25] Shan J, Ju Y (2009). A single-step synthesis and the kinetic mechanism for monodisperse and hexagonal-phase NaYF4:Yb Er upconversion nanophosphors. Nanotechnology.

[26] Chen B, Kong W, Wang N, Zhu G, Wang F (2019). Oleylamine-mediated synthesis of small NaYbF4 nanoparticles with tunable size. Chem. Mater..

[27] Boyer J-C, Vetrone F, Cuccia LA, Capobianco JA (2006). Synthesis of colloidal upconverting NaYF4 nanocrystals doped with Er3+, Yb3+ and Tm3+, Yb3+ via thermal decomposition of lanthanide trifluoroacetate precursors. J. Am. Chem. Soc..

[28] Mai H-X, Zhang Y-W, Sun L-D, Yan C-H (2007). Size- and phase-controlled synthesis of monodisperse NaYF4:Yb, Er nanocrystals from a unique delayed nucleation pathway monitored with upconversion spectroscopy. J. Phys. Chem. C.

[29] Ehlert O, Thomann R, Darbandi M, Nann T (2008). A four-color colloidal multiplexing nanoparticle system. ACS Nano.

[30] Ye X (2010). Morphologically controlled synthesis of colloidal upconversion nanophosphors and their shape-directed self-assembly. Proc. Natl. Acad. Sci..

[31] Liu C, Wang H, Li X, Chen D (2009). Monodisperse, size-tunable and highly efficient β-NaYF4:Yb, Er(Tm) up-conversion luminescent nanospheres: Controllable synthesis and their surface modifications. J. Mater. Chem..

[32] Li D, Shao Q, Dong Y, Jiang J (2014). Phase-, shape- and size-controlled synthesis of NaYF_4_:Yb^3^^+^, Er^3^^+^ nanoparticles using rare-earth acetate precursors. J. Rare Earths.

[33] Qian H-S, Zhang Y (2008). Synthesis of hexagonal-phase core–shell NaYF_4_ nanocrystals with tunable upconversion fluorescence. Langmuir.

[34] Na H, Woo K, Lim K, Jang HS (2013). Rational morphology control of β-NaYF_4_:Yb, Er/Tm upconversion nanophosphors using a ligand, an additive, and lanthanide doping. Nanoscale.

[35] Wilhelm S (2015). Water dispersible upconverting nanoparticles: Effects of surface modification on their luminescence and colloidal stability. Nanoscale.

[36] Liu D (2016). Three-dimensional controlled growth of monodisperse sub-50 nm heterogeneous nanocrystals. Nat. Commun..

[37] Jurga N (2022). Influence of the synthesis route on the spectroscopic, cytotoxic, and temperature-sensing properties of oleate-capped and ligand-free core/shell nanoparticles. J. Colloid Interface Sci..

[38] Hühn J (2017). Selected standard protocols for the synthesis, phase transfer, and characterization of inorganic colloidal nanoparticles. Chem. Mater..

[39] Xiao DL (2021). Advances and challenges of fluorescent nanomaterials for synthesis and biomedical applications. Nanoscale Res. Lett..

[40] Donega CD, Liljeroth P, Vanmaekelbergh D (2005). Physicochemical evaluation of the hot-injection method, a synthesis route for monodisperse nanocrystals. Small.

[41] Mir IA, Das K, Rawat K, Bohidar HB (2016). Hot injection versus room temperature synthesis of CdSe quantum dots: A differential spectroscopic and bioanalyte sensing efficacy evaluation. Colloids Surf. A Physicochem. Eng. Asp..

[42] Liu YJ (2022). Recent advances in scalable synthesis and performance of Janus polymer/inorganic nanocomposites. Prog. Mater. Sci..

[43] Zhang X (2019). Mass production of poly(ethylene glycol) monooleate-modified core-shell structured upconversion nanoparticles for bio-imaging and photodynamic therapy. Sci. Rep..

[44] Jiao Y (2020). Controllable synthesis of upconversion nanophosphors toward scale-up productions. Part. Part. Syst. Charact..

[45] Volk AA, Epps RW, Abolhasani M (2021). Accelerated development of colloidal nanomaterials enabled by modular microfluidic reactors: Toward autonomous robotic experimentation. Adv. Mater..

[46] Khizar S, Zine N, Errachid A, Jaffrezic-Renault N, Elaissari A (2022). Microfluidic-based nanoparticle synthesis and their potential applications. Electrophoresis.

[47] Jiao M, Zeng J, Jing L, Liu C, Gao M (2015). Flow Synthesis of biocompatible Fe_3_O_4_ nanoparticles: Insight into the effects of residence time, fluid velocity, and tube reactor dimension on particle size distribution. Chem. Mater..

[48] Pu Y, Cai F, Wang D, Wang J-X, Chen J-F (2018). Colloidal synthesis of semiconductor quantum dots toward large-scale production: A review. Ind. Eng. Chem. Res..

[49] Chan EM (2015). Combinatorial approaches for developing upconverting nanomaterials: High-throughput screening, modeling, and applications. Chem. Soc. Rev..

[50] Sebastian V, Arruebo M, Santos HA, Liu D, Zhang H (2019). Microfluidics for Pharmaceutical Applications.

[51] You W (2018). Large-scale synthesis of uniform lanthanide-doped NaREF_4_ upconversion/downshifting nanoprobes for bioapplications. Nanoscale.

[52] May PB, Suter JD, May PS, Berry MT (2016). The Dynamics of nanoparticle growth and phase change during synthesis of β-NaYF4. J. Phys. Chem. C.

[53] Radunz S (2018). Evolution of size and optical properties of upconverting nanoparticles during high-temperature synthesis. J. Phys. Chem. C.

[54] Radunz S (2019). Simple self-referenced luminescent pH sensors based on upconversion nanocrystals and pH-sensitive fluorescent BODIPY dyes. Anal. Chem..

[55] You M (2015). Inkjet printing of upconversion nanoparticles for anti-counterfeit applications. Nanoscale.

[56] Andresen E, Würth C, Prinz C, Michaelis M, Resch-Genger U (2020). Time-resolved luminescence spectroscopy for monitoring the stability and dissolution behaviour of upconverting nanocrystals with different surface coatings. Nanoscale.

[57] Kraft M, Würth C, Muhr V, Hirsch T, Resch-Genger U (2018). Particle-size-dependent upconversion luminescence of NaYF4:Yb, Er nanoparticles in organic solvents and water at different excitation power densities. Nano Res..

[58] Hao S (2014). Tuning the size and upconversion emission of NaYF4:Yb^3^^+^/Pr^3^^+^ nanoparticles through Yb^3^^+^ doping. RSC Adv..

[59] Chen B, Wang F (2020). Recent advances in the synthesis and application of Yb-based fluoride upconversion nanoparticles. Inorg. Chem. Front..

[60] Würth C, Fischer S, Grauel B, Alivisatos AP, Resch-Genger U (2018). Quantum yields, surface quenching, and passivation efficiency for ultrasmall core/shell upconverting nanoparticles. J. Am. Chem Soc..

[61] Wang F (2010). Simultaneous phase and size control of upconversion nanocrystals through lanthanide doping. Nature.

[62] Homann C (2018). NaYF_4_:Yb, Er/NaYF_4_ core/shell nanocrystals with high upconversion luminescence quantum yield. Angew. Chem. Int. Ed..

[63] Würth C (2022). Yb- and Er concentration dependence of the upconversion luminescence of highly doped NaYF4:Yb, Er/NaYF4:Lu core/shell nanocrystals prepared by a water-free synthesis. Nano Res..

[64] Kaiser M (2017). Power-dependent upconversion quantum yield of NaYF4:Yb^3^^+^, Er^3^^+^ nano- and micrometer-sized particles—Measurements and simulations. Nanoscale.

[65] Wurth C (2017). Excitation power dependent population pathways and absolute quantum yields of upconversion nanoparticles in different solvents. Nanoscale.

