# Supplementary material for: Assessing the reproducibility and up-scaling of the synthesis of Er,Yb-doped NaYF4-based upconverting nanoparticles and control of size, morphology, and optical properties
Source: Sci Rep. 2023 Feb 9;13:2288. doi: 10.1038/s41598-023-28875-8 (PMC9911732; doi:10.1038/s41598-023-28875-8)
Supplement: Supplementary file 1 — Supplementary Information. [file 41598_2023_28875_MOESM1_ESM.pdf]

Supporting Information

Assessing the Reproducibility and Up-Scaling of Er,Yb-doped $NaYF_4$-based Upconverting Nanoparticles and Control of the Size, Morphology, and Optical Properties

Elina Andresen[1,*], Fahima Islam[1], Carsten Prinz[2], Philipp Gehrmann[3], Kai Licha[3], Janina Roik[4], Sebastian Recknagel[4], and Ute Resch-Genger[1,*]

[1] Federal Institute of Materials Research and Testing (BAM), Division *Biophotonics*, Richard-Willstätter-Str. 11, 12489 Berlin, Germany

[2] Federal Institute of Materials Research and Testing (BAM), Division *Structure Analytics*, Richard-Willstätter-Str. 11, 12489 Berlin, Germany

[3] FEW Chemicals GmbH, Technikumstraße 1, 06766 Bitterfeld-Wolfen

[4] Federal Institute of Materials Research and Testing (BAM), Division *Inorganic Reference Materials*, Richard-Willstätter-Str. 11, 12489 Berlin, Germany

*elina.andresen@bam.de

*ute.resch@bam.de

Table of Contents

1. List of the performed synthesis and nomenclature of the samples 2
2. Overview of the reaction conditions 3
3. TEM images and histograms of all samples 4
4. ICP-OES data 13
5. Calculated Red-to-Green ratio and intensity weighted lifetimes of the emissive states of all samples 14
6. Optical characterization of the samples from the reproducibility, scale-up, and robustness series 15
7. X-ray diffraction (XRD) patterns of the samples from the dopant concentration series 17
8. Optical characterization of the samples from the dopant concentration series 18
9. Synthesis of UCNPs starting from low-price Ln-precursors 19

1. List of the performed synthesis and nomenclature of the samples

Table S1: List of the performed synthesis and nomenclature of the obtained samples

Series Name	Sample composition	Reaction time and temperature	Solvent composition	Variable parameter	Sample name
**Reproducibility**	$NaYF_4$: 20 % Yb^{3+}, 2 % Er^{3+}	30 min (325°C)	40 ml OA : 80 ml ODE	–	**STD 1 – STD 10**
**Robustness**	$NaYF_4$: 20 % Yb^{3+}, 2 % Er^{3+}	30 min (325°C),<br>60 min (325°C),<br>30 min (335°C)	40 ml OA : 80 ml ODE	**Temperature**<br>**Time**	STD 1<br>**STD 1_60min**<br>**STD 1_335°C**
**Scale up**	$NaYF_4$: 20 % Yb^{3+}, 2 % Er^{3+}	30 min (325°C)	20 ml OA: 40 ml ODE<br>40 ml OA : 80 ml ODE<br>80 ml OA : 160 ml ODE<br>200 ml OA : 400 ml ODE	**Batch size**<br>0.5x<br>1x<br>2x<br>5x	**STD 1_0.5x**<br>STD 1<br>**STD 1_2x**<br>**STD 1_5x**
**Solvent influence**	$NaYF_4$: 20 % Yb^{3+}, 2 % Er^{3+}	60 min (325°C)	50 ml OA : 70 ml ODE<br>40 ml OA : 80 ml ODE<br>30 ml OA : 90 ml ODE<br>20 ml OA: 100 ml ODE	**Ratio OA : ODE**<br>5:7<br>4:8<br>3:9<br>2:10	**OA-50**<br>OA-40 (STD 1_60 min)<br>**OA-30**<br>**OA-20**
**Dopant influence**	$NaYF_4$: x % Yb^{3+}, 2 % Er^{3+}<br>(x = 20, 40, 60, 70)<br>$NaYbF_4$: 2 % Er^{3+}	30 min (325°C)	40 ml OA: 80 ml ODE	**Yb^{3+} concentration**<br>20 mol%<br>40 mol%<br>60 mol%<br>70 mol%<br>98 mol%	Yb-20 (STD 1)<br>**Yb-40**<br>**Yb-60**<br>**Yb-70**<br>**Yb-98**
**Precursor concentration influence**	$NaYF_4$: 20 % Yb^{3+}, 2 % Er^{3+}	60 min (325°C),<br>90 min (325°C)	40 ml OA : 80 ml ODE	**Precursor concentration**<br>5 mmol<br>10 mmol<br>15 mmol (t=60 min)<br>15 mmol (t=90 min)	Conc-5 (STD 1_60 min)<br>**Conc-10**<br>**Conc-15**<br>**Conc-15_90min**

2. Overview of the reactant amounts

Table S2: Overview of the reactant amounts used during the synthesis

	$YCl_3·6H_2O$	$YbCl_3·6H_2O$	$ErCl_3·6H_2O$	NaOH	NH_4F	Oleic acid	Octadecene
	mg (mmol)	mg (mmol)	mg (mmol)	mg (mmol)	mg (mmol)	mL	mL
**STD 1 – STD 10**	1183.3 mg (3.90 mmol)	391.7 mg (1.01 mmol)	39.90 mg (0.10 mmol)	519.1 mg (12.98 mmol)	742.4 mg (20.04 mmol)	40	80
**STD 1_60min**	1183.3 mg (3.90 mmol)	391.7 mg (1.01 mmol)	39.90 mg (0.10 mmol)	519.1 mg (12.98 mmol)	742.4 mg (20.04 mmol)	40	80
**STD 1_335°C**	1183.5mg (3.90mmol)	387.4 mg (1 mmol)	38.2mg (0.1 mmol)	511 mg (12.78 mmol)	740.8 mg (20 mmol)	40	80
**STD 1_0.5x**	591.75 mg (1.95 mmol)	193.8 mg (0.5 mmol)	16.0 mg (0.05 mmol)	259.3 mg (6.48 mmol)	370.3 mg (10.01 mmol)	20	40
**STD 1_2x**	2366.21mg (7.80mmol)	774.98 mg (2 mmol)	76.34 mg (0.20 mmol)	1000 mg (25 mmol)	1481 mg (39.98 mmol)	80	160
**STD 1_5x**	5920.2 mg (19.52 mmol)	1939.6 mg (5.01 mmol)	192.1 mg (0.50 mmol)	2527.1 mg (63.18 mmol)	3704.0 mg (100 mmol)	200	390
**OA-50**	1182.1 mg (3.90 mmol)	387.1mg (1 mmol)	38.5 mg (0.10 mmol)	512 mg (12.8 mmol)	740 mg (19.98 mmol)	50	70
**OA-30**	1183.1 mg (3.90 mmol)	387.49mg (1 mmol)	38.17 mg (0.10 mmol)	518.7 mg (12.97 mmol)	741.4 mg (20.02 mmol)	30	90
**OA-20**	1183.4 mg (3.90 mmol)	487.7mg (1.26 mmol)	38.4 mg (0.10 mmol)	518.7 mg (12.97 mmol)	740.7 mg (20 mmol)	20	100
**Yb-40**	439.9 mg (1.45 mmol)	388.4mg (1 mmol)	19.2 mg (0.05 mmol)	258.4 mg (6.46 mmol)	387 mg (10.21 mmol)	20	40
**Yb-60**	576.4 mg (1.90 mmol)	1162.8mg (3 mmol)	38.2 mg (0.10 mmol)	515 mg (12.88 mmol)	741.4 mg (20.02 mmol)	40	80
**Yb-70**	84.9 mg (0.28 mmol)	271.6 mg (0.70 mmol)	7.63 mg (0.20 mmol)	101.60 mg (2.54 mmol)	148.20 mg (4 mmol)	6	15
**Yb-98**	- -	1905.6 mg (4.92 mmol)	39.7 mg (0.10 mmol)	508.4 mg (12.71 mmol)	746.4 mg (20.15 mmol)	40	80
**Conc-10**	2370.3 mg (7.81 mmol)	779.3 mg (2.01 mmol)	78.0 mg (0.20 mmol)	1037.0 mg (25.93 mmol)	1483.0 mg (40.04 mmol)	40	80
**Conc-15**	3577.4 mg (11.79 mmol)	1189.7 mg (3.07 mmol)	119.3 mg (0.31 mmol)	1548.3 mg (38.71 mmol)	2222.0 mg (59.99 mmol)	40	80
**Conc-15_90min**	3569.8 mg (11.77 mmol)	1228.6 mg (3.17 mmol)	120.1 mg (0.31 mmol)	1535.2 mg (38.38 mmol)	2227.6 mg (60.14 mmol)	40	80

3. TEM images and histograms of all samples

a)

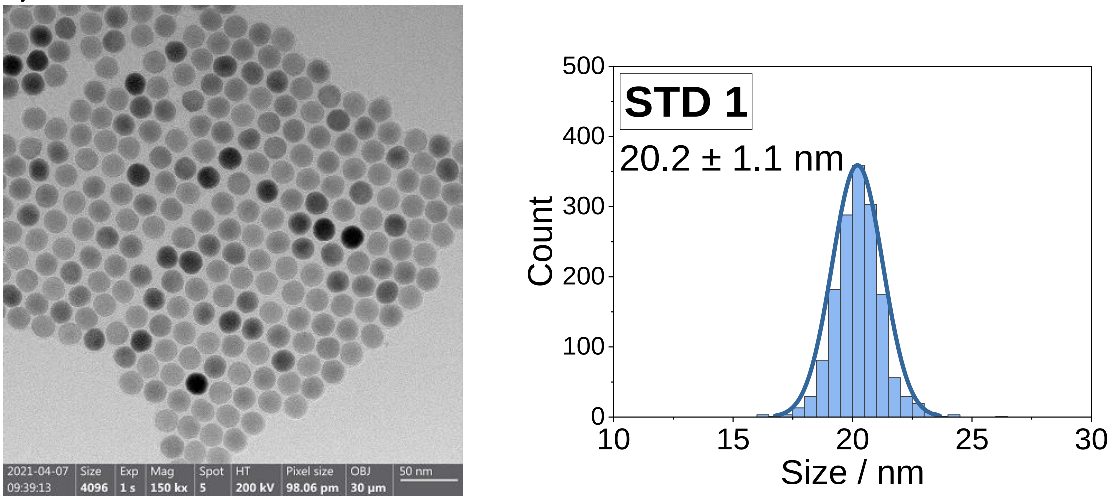

b)

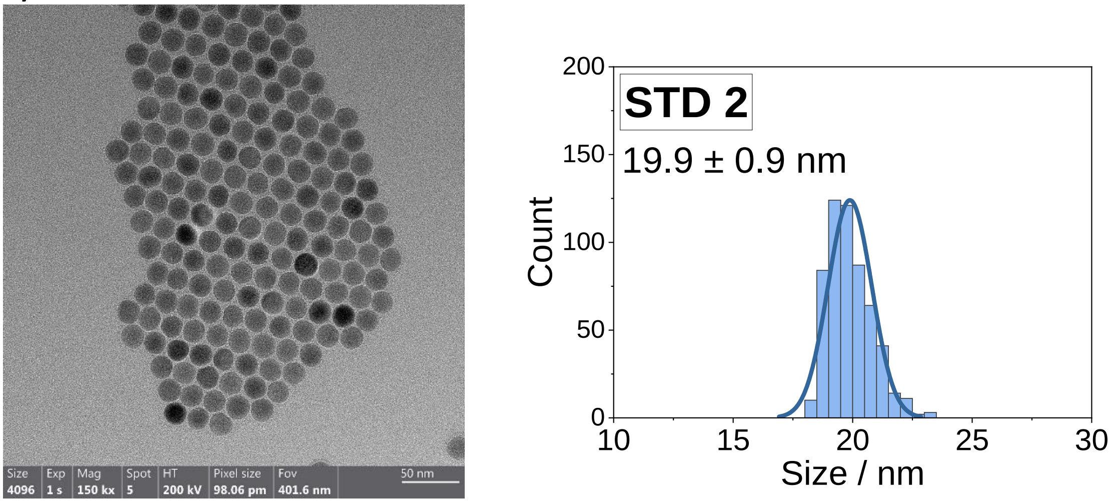

c)

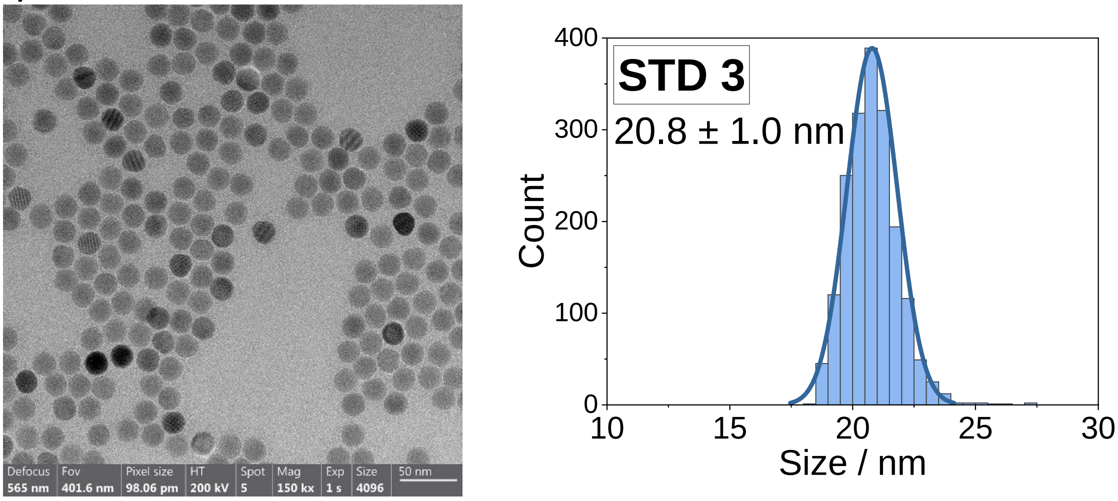

Figure S1: continued next page

d)

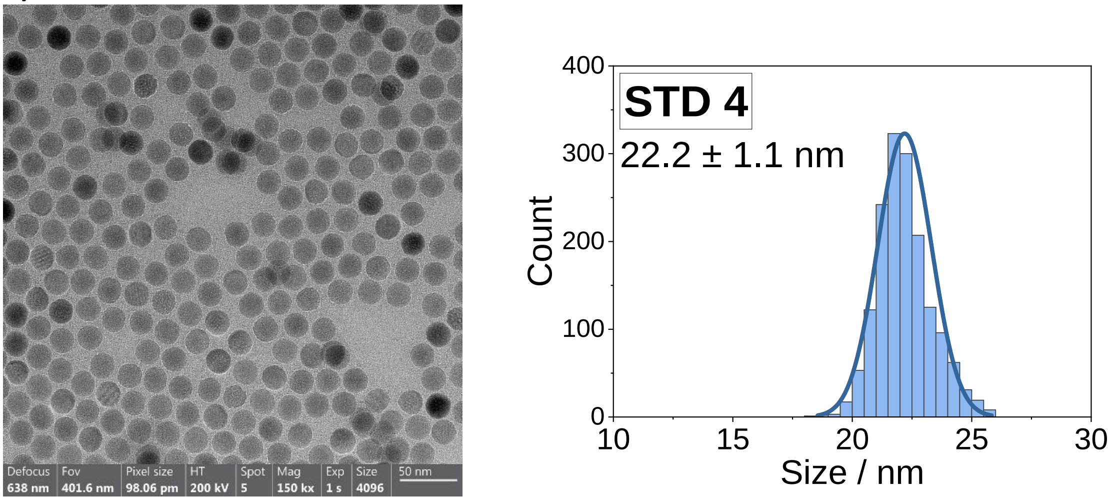

e)

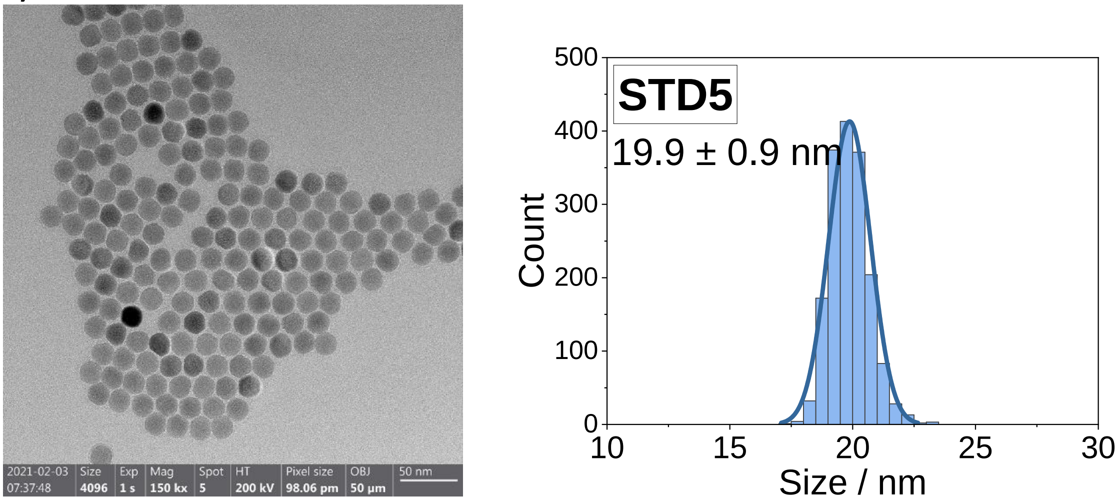

f)

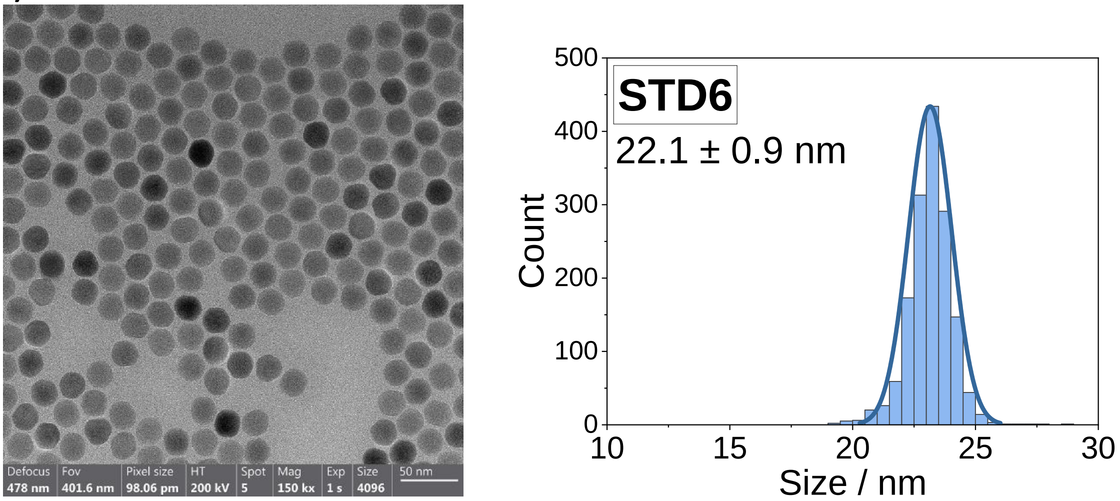

Figure S1: continued next page

g)

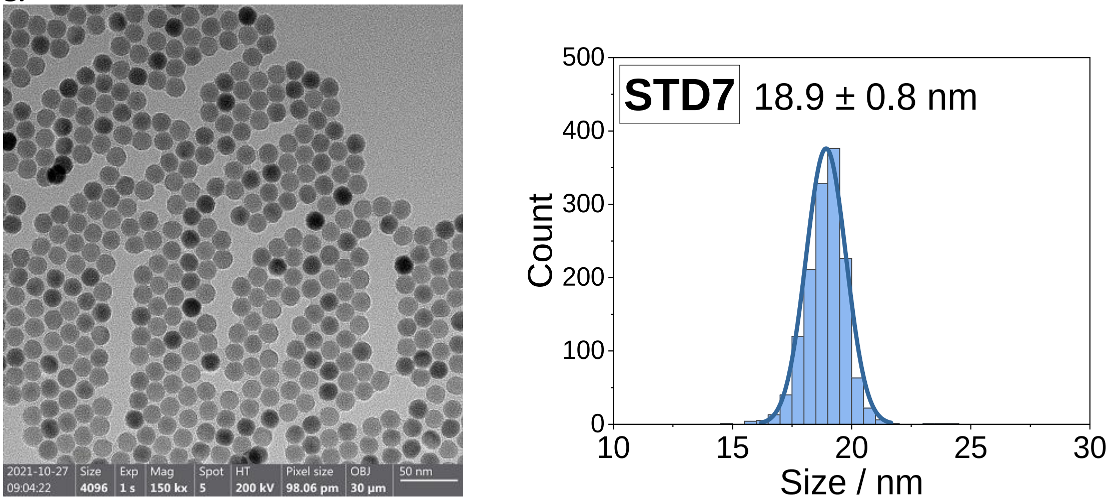

h)

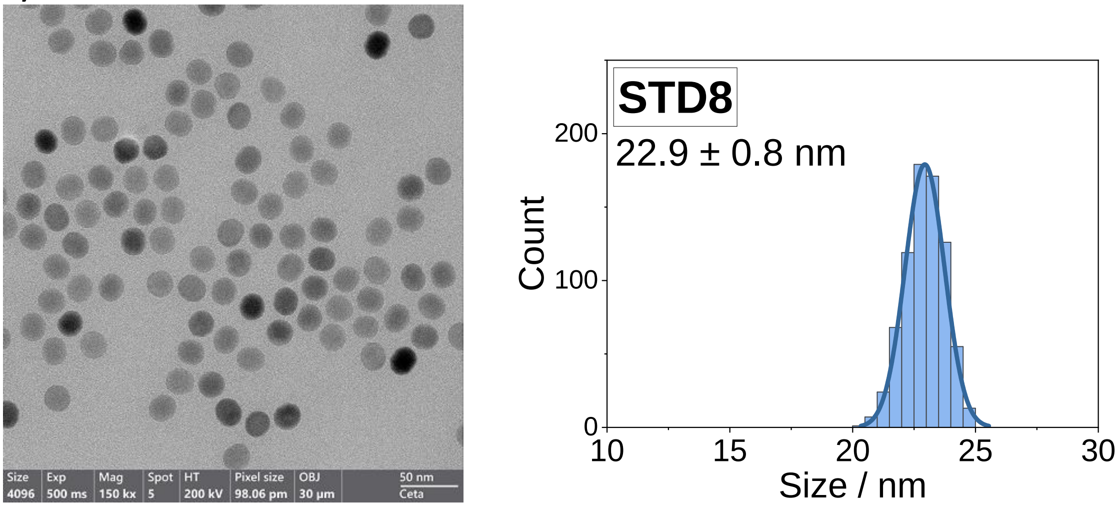

i)

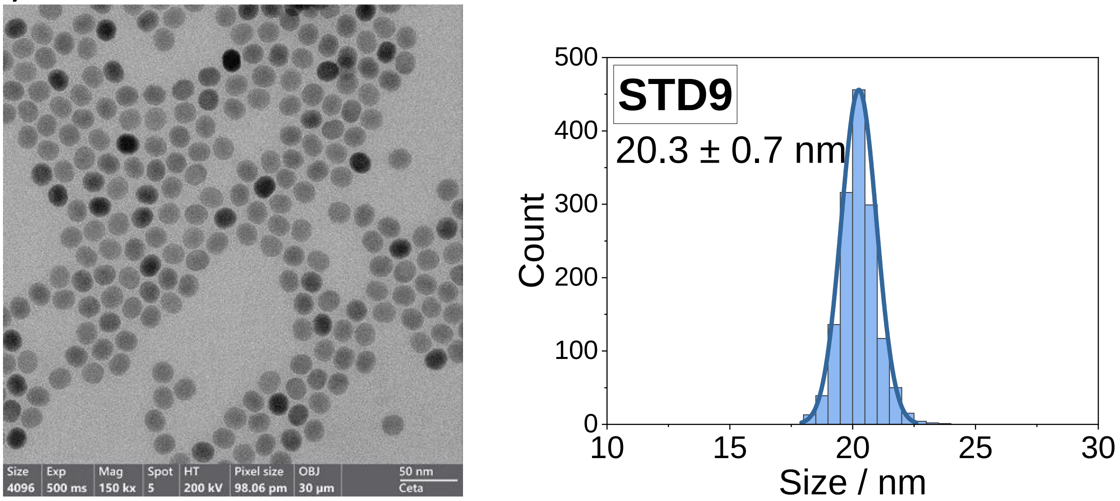

Figure S1: continued next page

j)

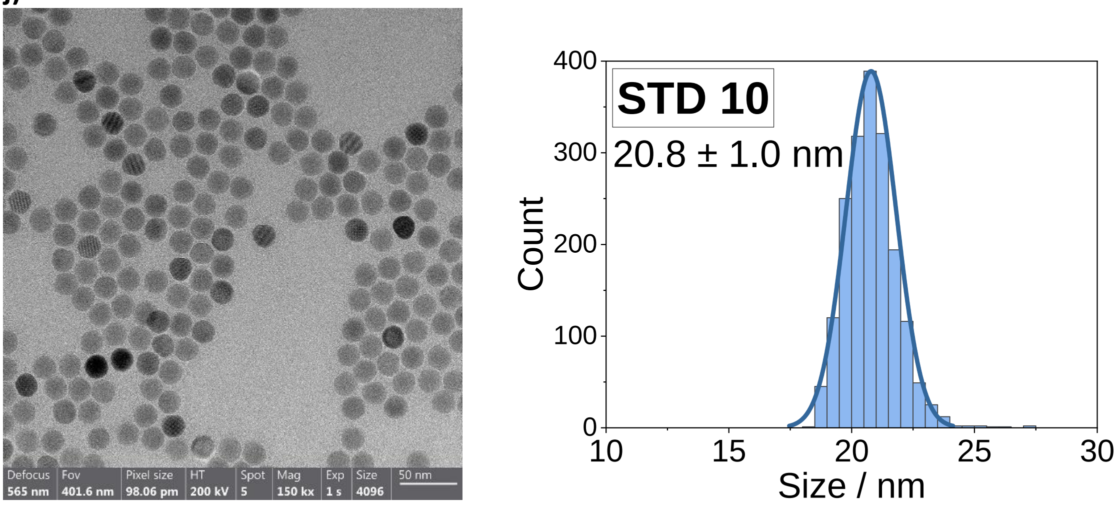

k)

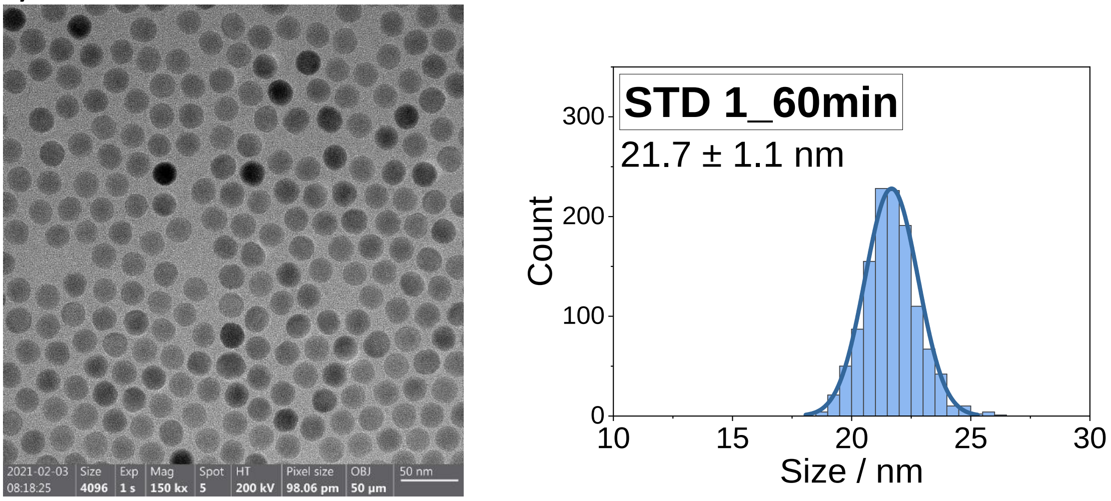

l)

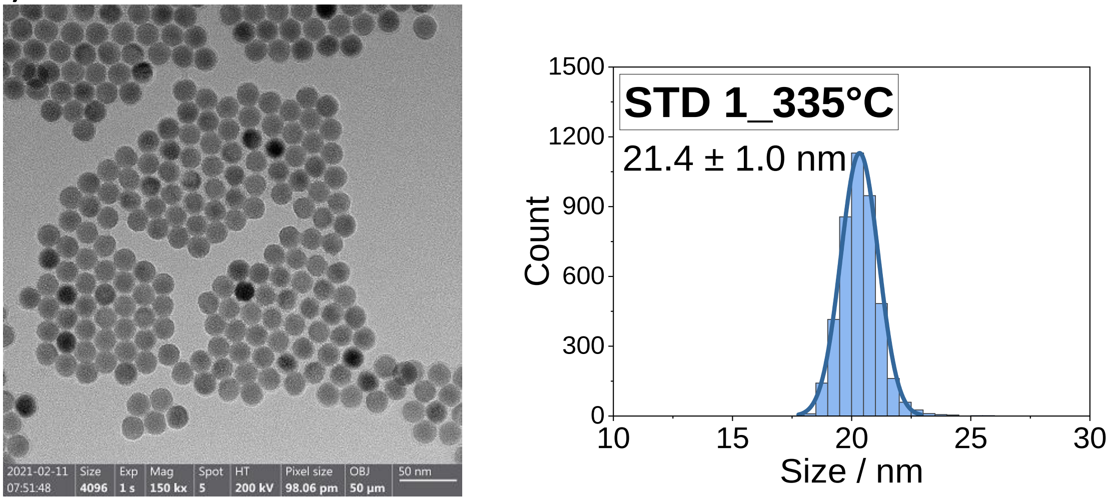

Figure S1: continued next page

m)

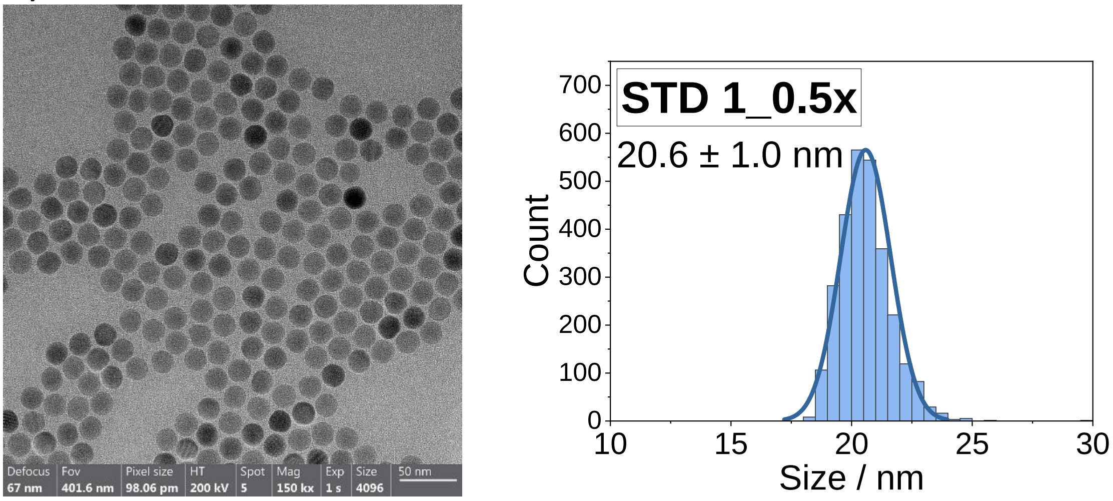

n)

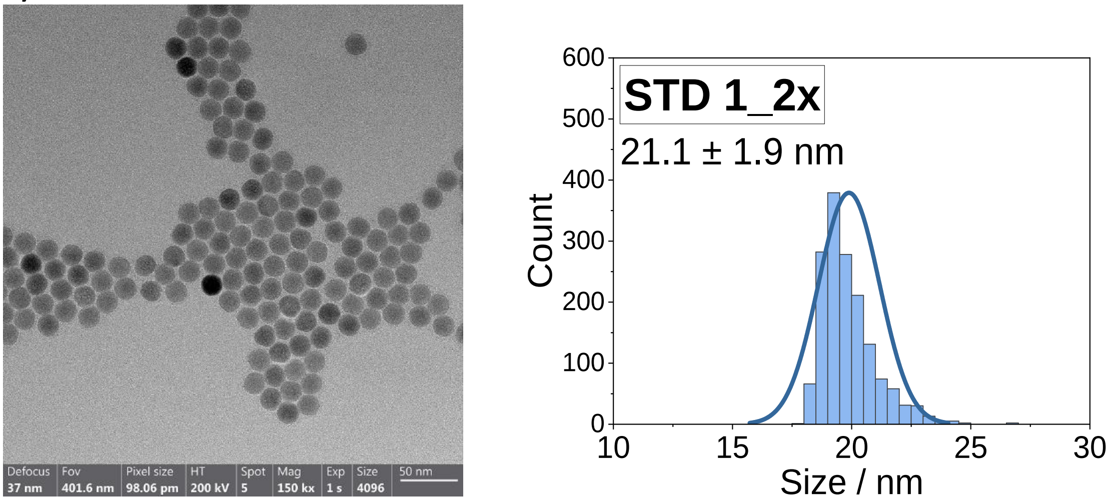

o)

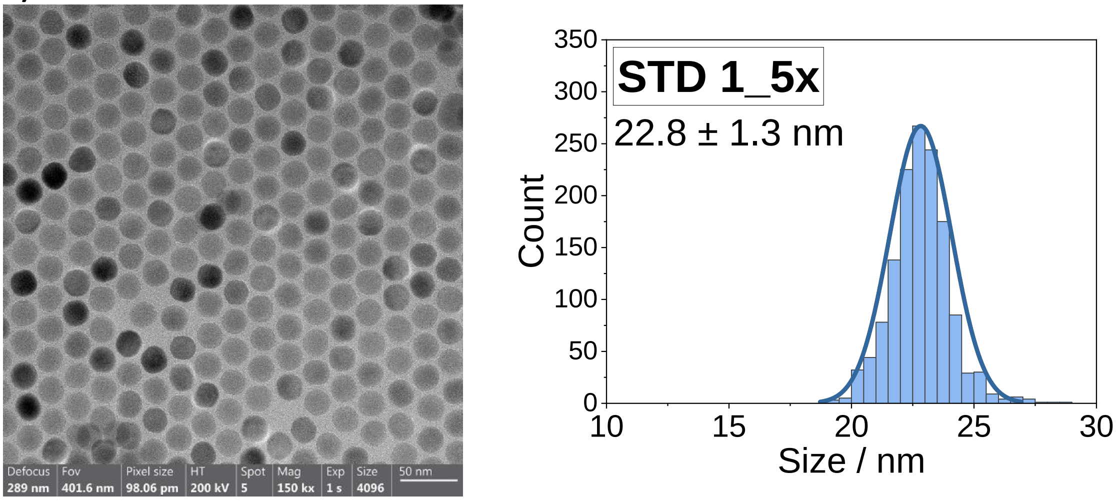

Figure S1: continued next page

p)

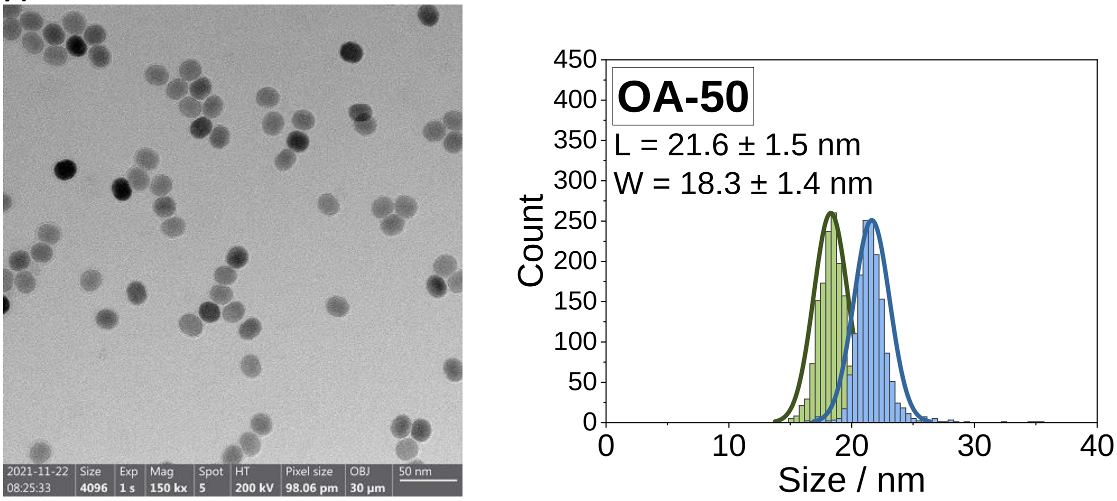

q)

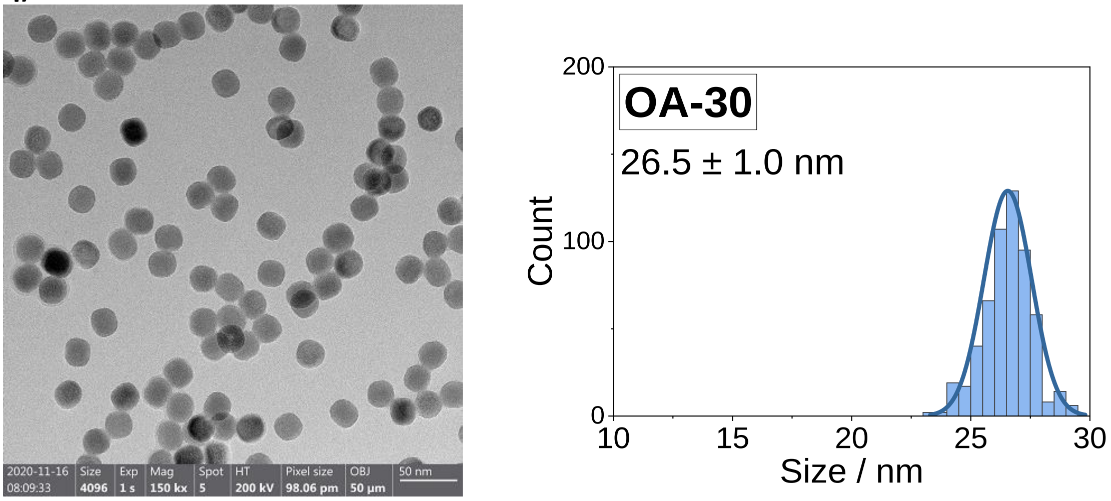

r)

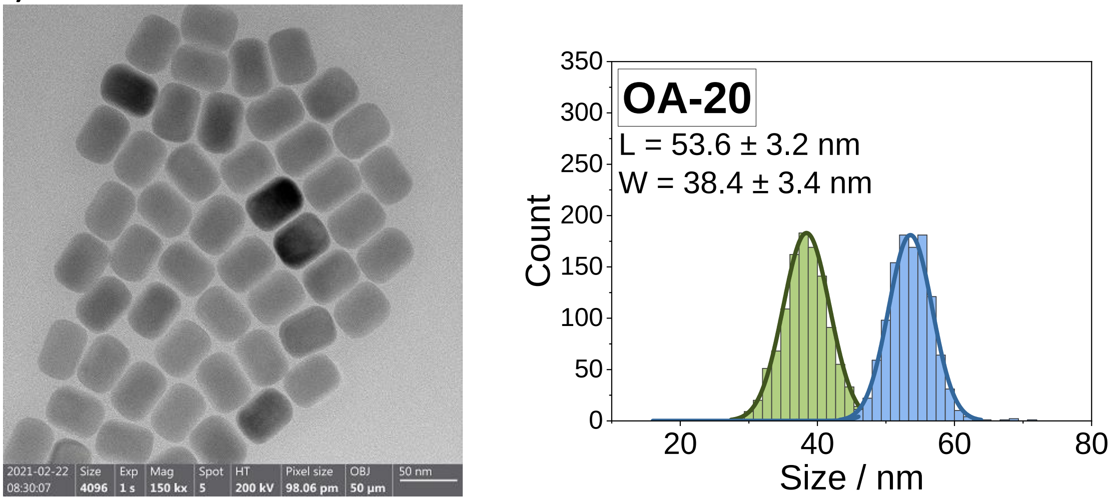

Figure S1: continued next page

s)

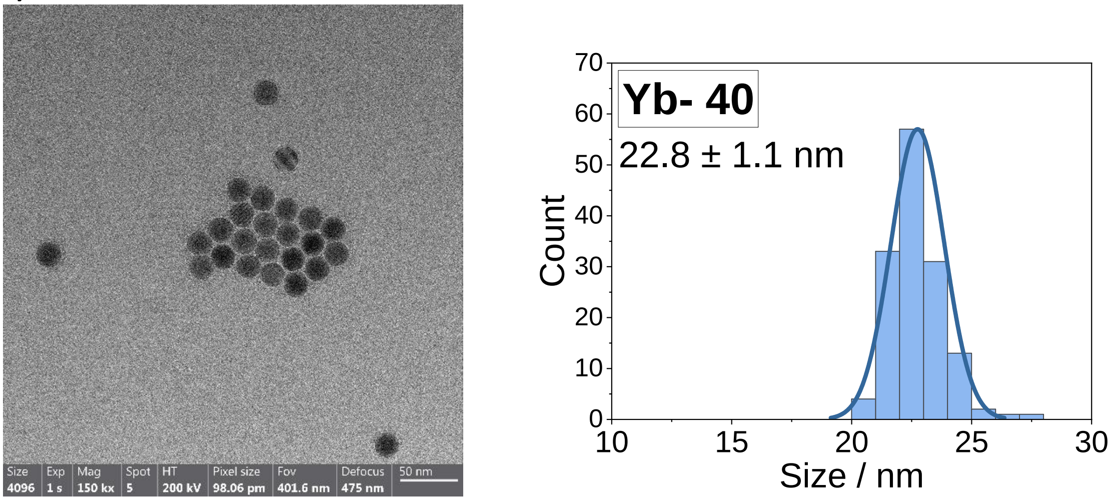

t)

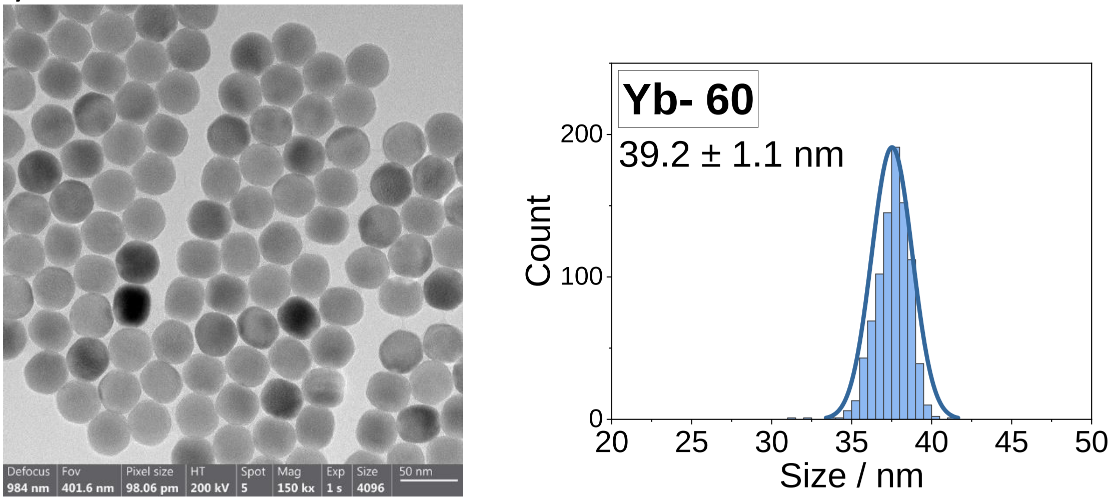

u)

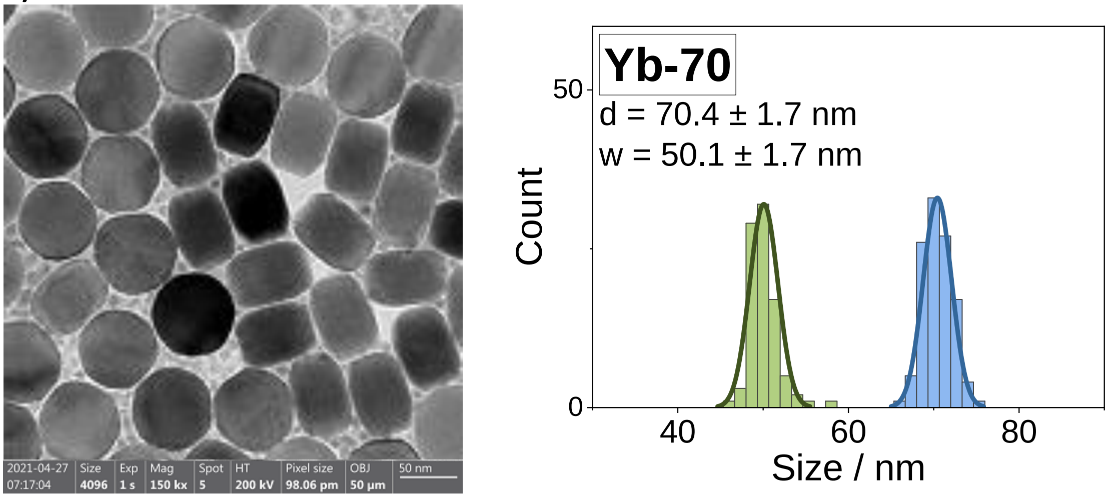

Figure S1: continued next page

v)

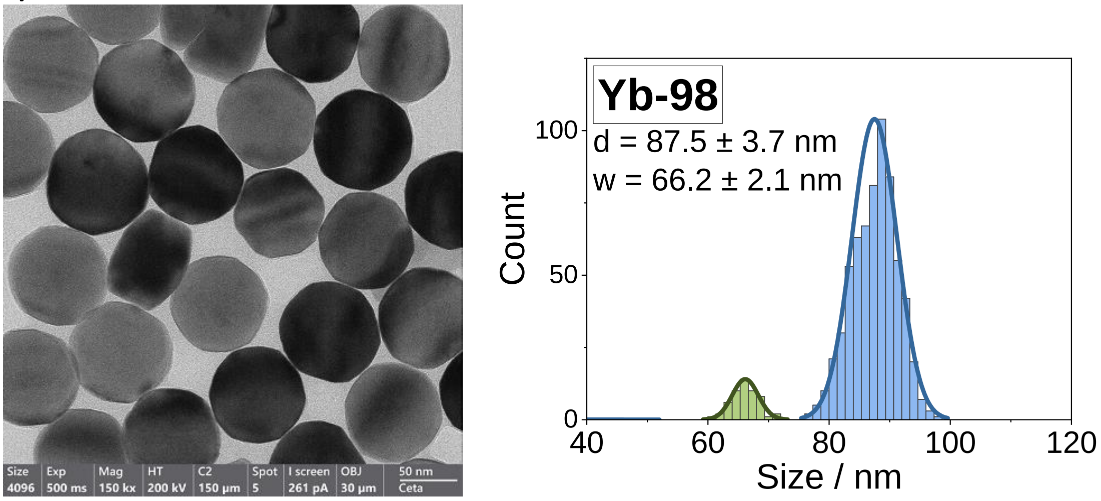

w)

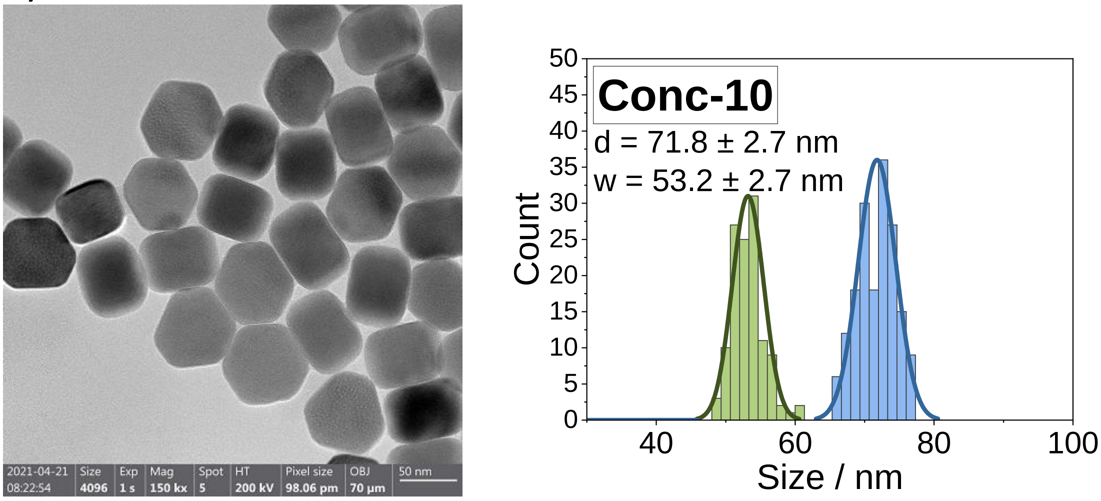

x)

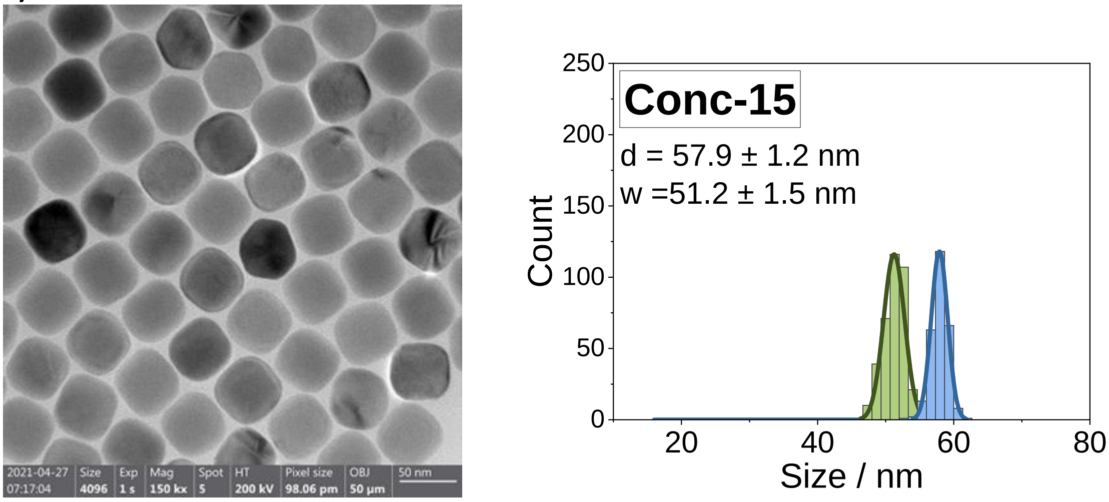

Figure S1: continued next page

y)

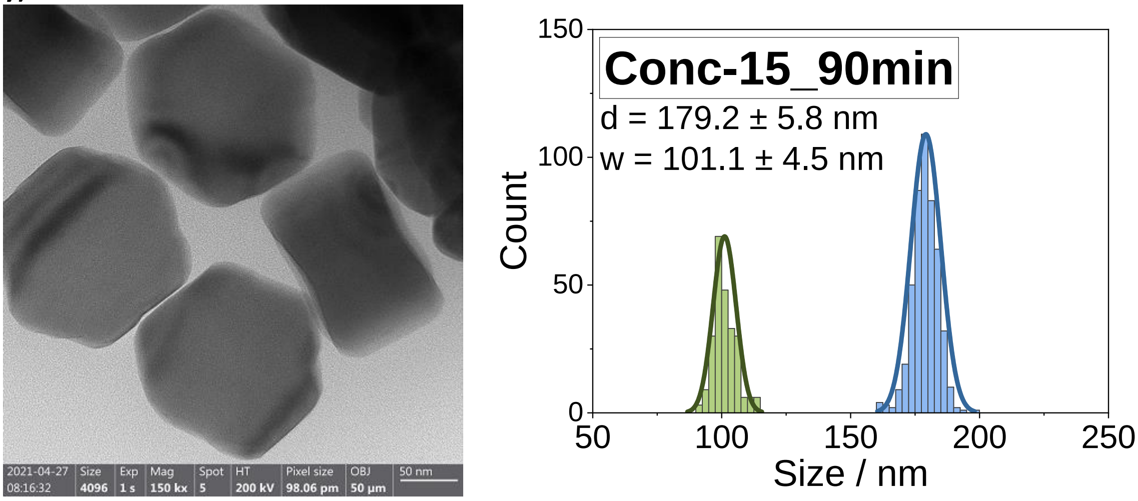

z)

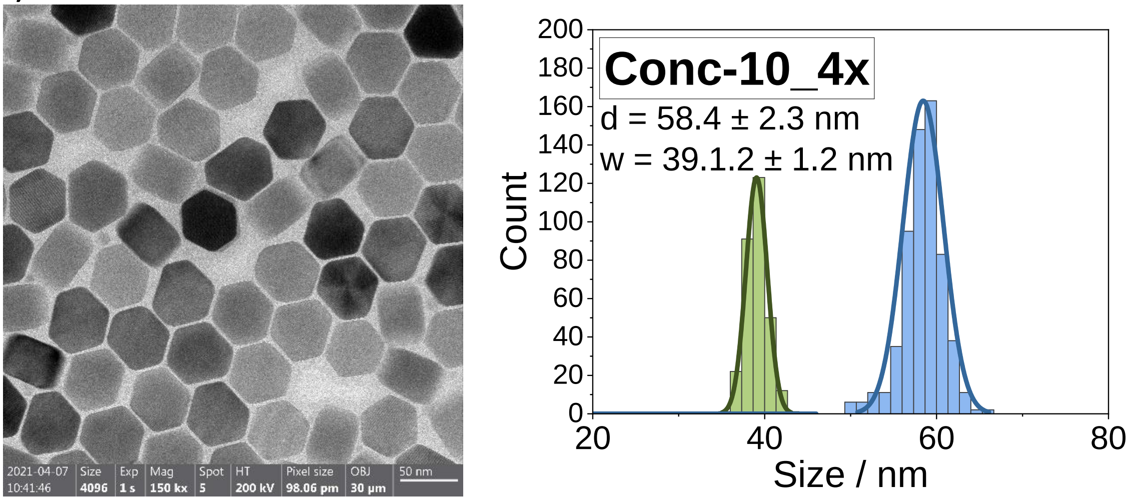

Figure S1: TEM images of as-synthesized oleate-$NaYF_4$:Yb,Er (magnification 150 kx) with the corresponding size histograms derived from TEM data (size distribution was fitted applying a Gaussian normal distribution)

4. ICP-OES Data

Table S3: Material composition (dopant concentration) of the synthesized UCNPs determined by ICP-OES measurements

	Expected /mol%			Determined by ICP-OES /mol%		
**Sample name**	Y^{3+}	Yb^{3+}	Er^{3+}	Y^{3+}	Yb^{3+}	Er^{3+}
**STD 1**	78	20	2	78.65 ± 1.66	19.30 ± 0.37	2.05 ± 0.03
**STD 2**	78	20	2	78.69 ± 0.47	19.27 ± 0.18	2.04 ± 0.01
**STD 3**	78	20	2	78.72 ± 1.74	19.25 ± 0.35	2.04 ± 0.03
**STD 4**	78	20	2	78.24 ± 0.65	19.62 ± 0.25	2.13 ± 0.01
**STD 5**	78	20	2	78.68 ± 1.11	19.35 ± 0.30	1.98 ± 0.03
**STD 6**	78	20	2	78.95 ± 0.66	19.01 ± 0.23	2.04 ± 0.02
**STD 7**	78	20	2	79.00 ± 1.83	18.98 ± 0.31	2.02 ± 0.03
**STD 8**	78	20	2	78.50 ± 1.06	19.47 ± 0.23	2.02 ± 0.01
**STD 9**	78	20	2	79.03 ± 0.68	18.95 ± 0.16	2.02 ± 0.01
**STD 10**	78	20	2	79.07 ± 0.87	19.98 ± 0.19	1.96 ± 0.02
**STD 1_60min**	78	20	2	78.57 ± 0.87	19.38 ± 0.29	2.06 ± 0.02
**STD 1_335°C**	78	20	2	78.12 ± 0.60	19.85 ± 0.09	2.04 ± 0.01
**STD 1_0.5x**	78	20	2	78.53 ± 0.62	19.46 ± 0.44	2.01 ± 0.04
**STD 1_2x**	78	20	2	77.97 ± 1.36	20.01 ± 0.32	2.02 ± 0.03
**STD 1_5x**	78	20	2	78.52 ± 1.44	19.45 ± 0.28	2.02 ± 0.02
**OA-50**	78	20	2	78.51 ± 0.76	19.47 ± 0.16	2.02 ± 0.01
**OA-30**	78	20	2	78.53 ± 0.62	19.46 ± 0.44	2.01 ± 0.04
**OA-20**	78	20	2	77.95 ± 1.76	20.07 ± 0.57	1.98 ± 0.03
**Yb-40**	58	40	2	57.79 ± 1.09	40.27 ± 0.51	1.94 ± 0.04
**Yb-60**	38	60	2	37.54 ± 1.05	60.34 ± 1.08	2.13± 0.04
**Yb-70**	28	70	2	27.65 ± 0.92	70.21 ± 2.26	2.14± 0.05
**Yb-98**	0	98	2	---	97.85 ± 0.93	2.15 ± 0.05
**Conc-10**	78	20	2	78.91 ± 0.78	19.06 ± 0.19	2.03 ± 0.01
**Conc-15**	78	20	2	77.71 ± 1.62	20.23 ± 0.35	2.06 ± 0.03
**Conc-15_90min**	78	20	2	78.53 ± 0.62	19.46 ± 0.44	2.01 ± 0.04

5. Calculated Red-to-Green ratio and intensity weighted lifetimes of the emissive states of all samples

Table S4: Red-to-Green ratio determined from the emission spectra and intensity weighted lifetimes calculated according to eq. S1 of the emissive states of all samples

Sample name	Red-to-Green ratio	Time-resolved measurements	
		Decay 540 nm	Decay 654 nm
**STD 1**	0.35	93 µs	169 µs
**STD 2**	0.32	99 µs	191 µs
**STD 3**	0.27	95 µs	187 µs
**STD 4**	0.31	87 µs	154 µs
**STD 5**	0.28	87 µs	157 µs
**STD 6**	0.29	101 µs	170 µs
**STD 7**	0.28	79 µs	143 µs
**STD 8**	0.30	100 µs	200 µs
**STD 9**	0.24	90 µs	195 µs
**STD 10**	0.32	100 µs	171 µs
**STD 1_60min**	0.26	96 µs	184 µs
**STD 1_335°C**	0.29	87 µs	156 µs
**STD 1_0.5x**	0.30	89 µs	196 µs
**STD 1_2x**	0.25	89 µs	159 µs
**STD 1_5x**	0.37	89 µs	161 µs
**OA-50**	0.34	81 µs	148 µs
**OA-30**	0.34	94 µs	180 µs
**OA-20**	1.00	170 µs	307 µs
**Yb-40**	0.53	62 µs	133 µs
**Yb-60**	1.33	56 µs	264 µs
**Yb-70**	2.08	42 µs	348 µs
**Yb-98**	2.68	34 µs	366 µs
**Conc-10**	0.99	236 µs	430 µs
**Conc-15**	1.13	315 µs	483 µs
**Conc-15_90min**	0.86	500 µs	525 µs

Intensity weighted lifetime: $$\tau_{int} = \frac{A_1\tau_1^2 + A_2\tau_2^2}{A_1\tau_1 + A_2\tau_2}$$ **(eq. S3)**

6. Optical characterization of the samples from the Reproducibility, Scale-up, and Robustness series

a)

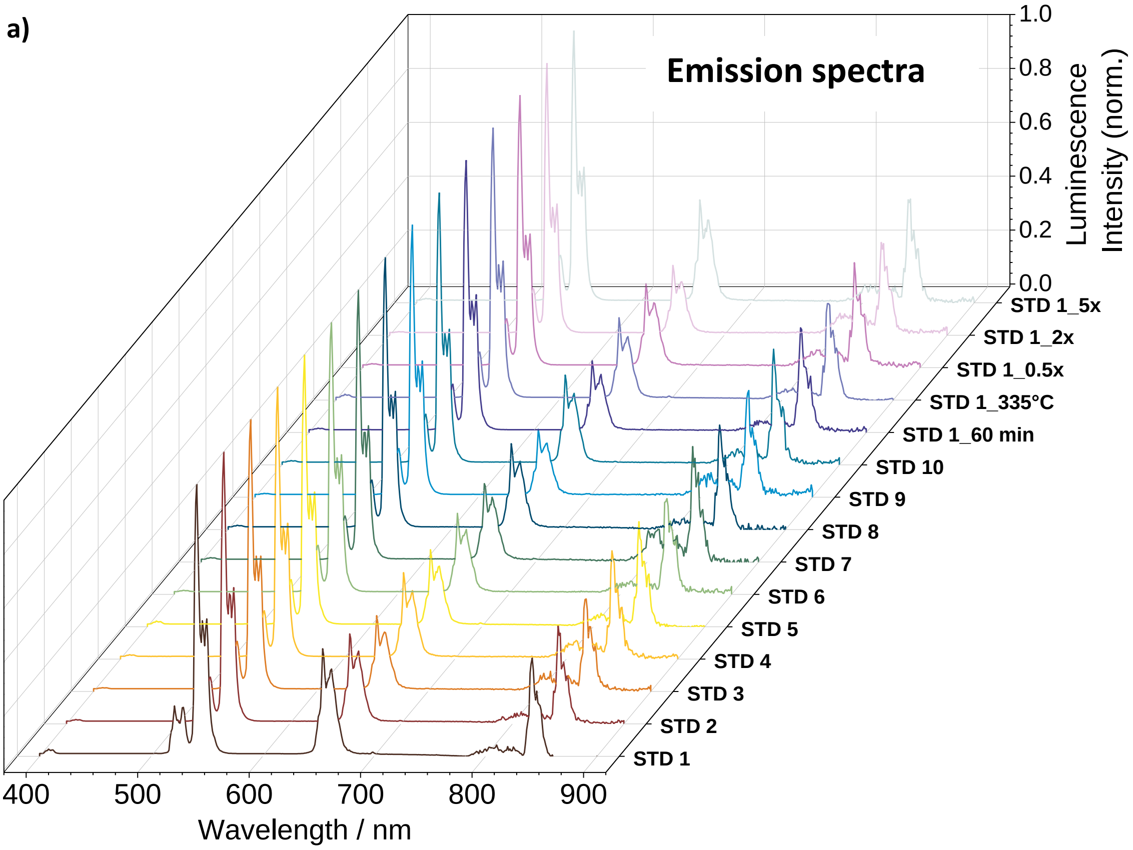

b)

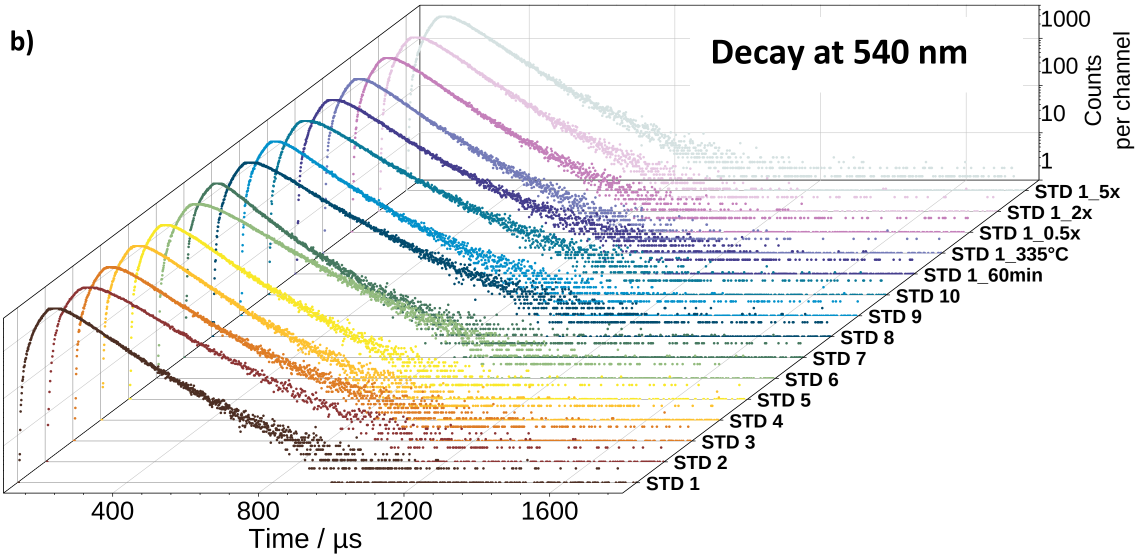

Figure S2: continued next page

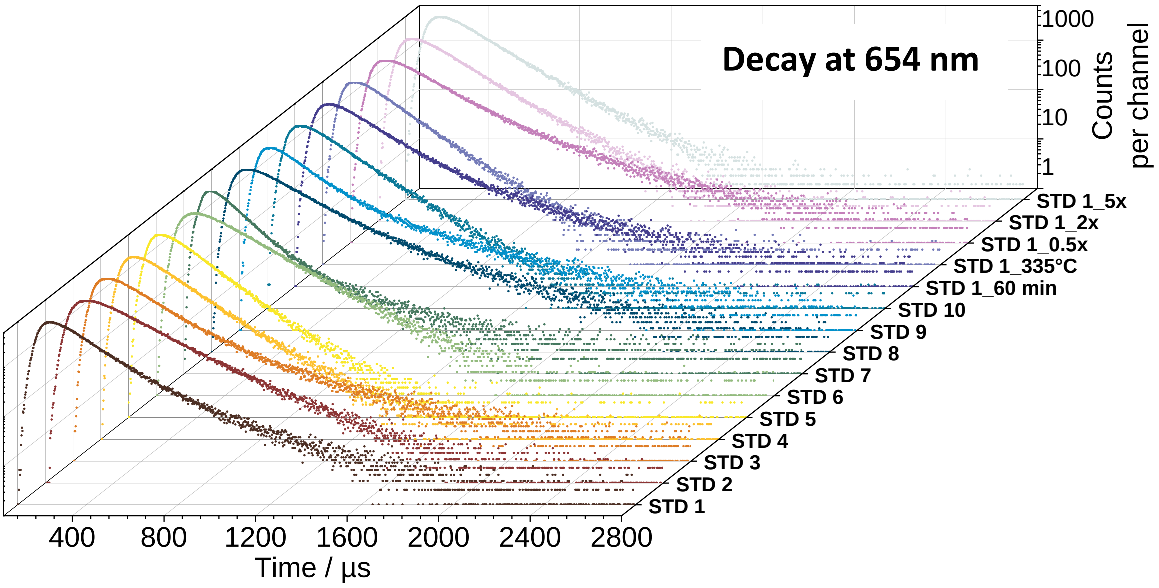

d)

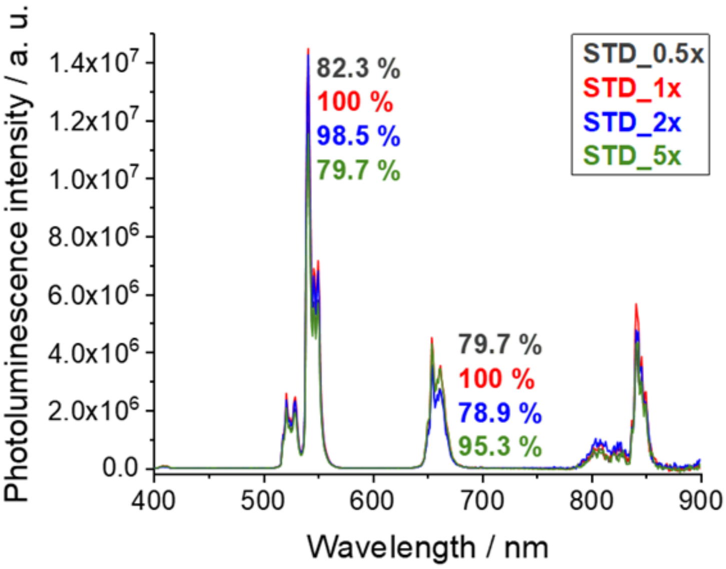

Figure S2: Optical characterization of the samples from the Reproducibility, Scale-up, and Robustness series: a) Normalized emission spectra; b) Decay curves at 540 nm; c) Decay curves at 654 nm and d) As measured photoluminescence spectra of the Scale-up series for sample concentration of 1 mg/mL.

7. X-ray diffraction (XRD) patterns of the samples from the dopant concentration series

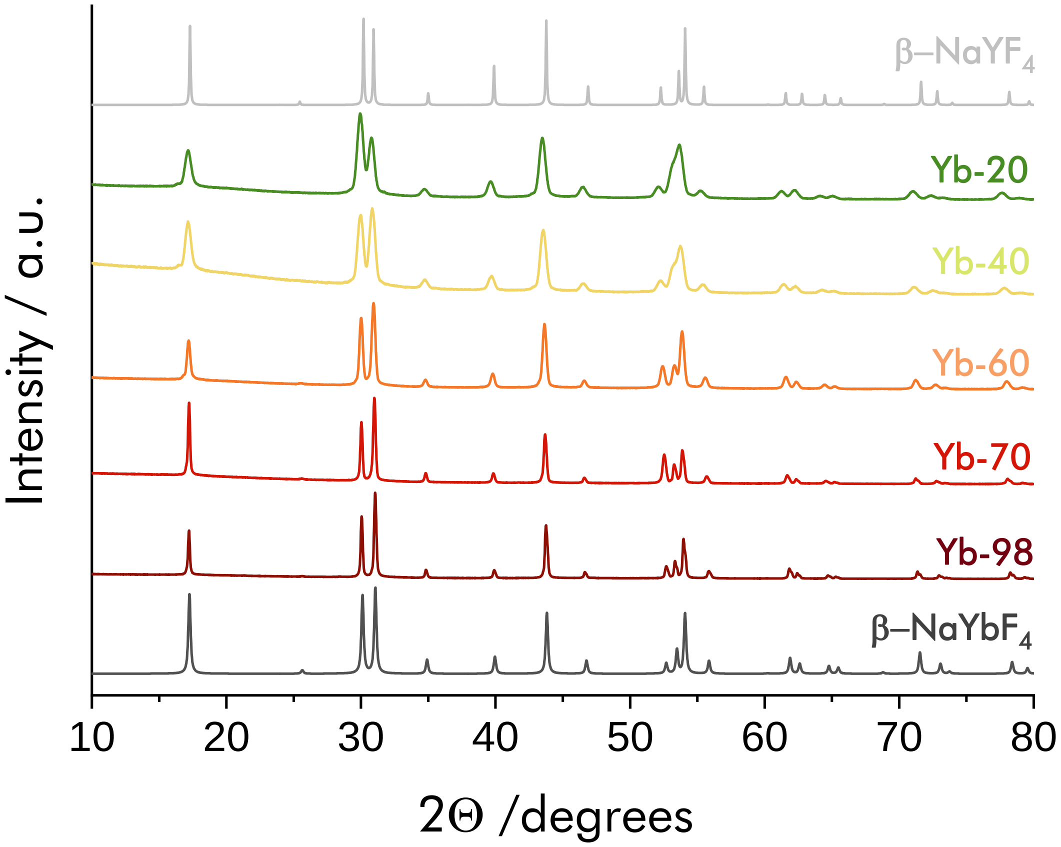

Figure S3: X-ray diffraction (XRD) patterns of samples **Yb-20**, **Yb-40**, **Yb-60**, **Yb-70** and **Yb-98** with references beta (β) phase $NaYF_4$ and beta (β) phase $NaYbF_4$.

8. Optical characterization of the samples from the dopant concentration series

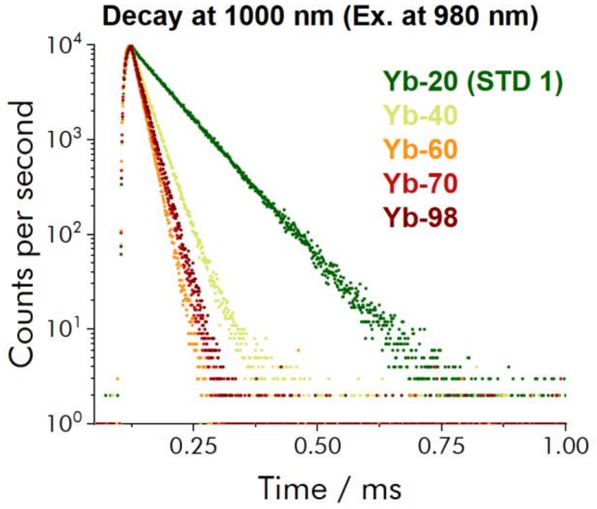

Figure S4: Decay kinetics of down-shifted emission of Yb^{3+} at 1000 nm.

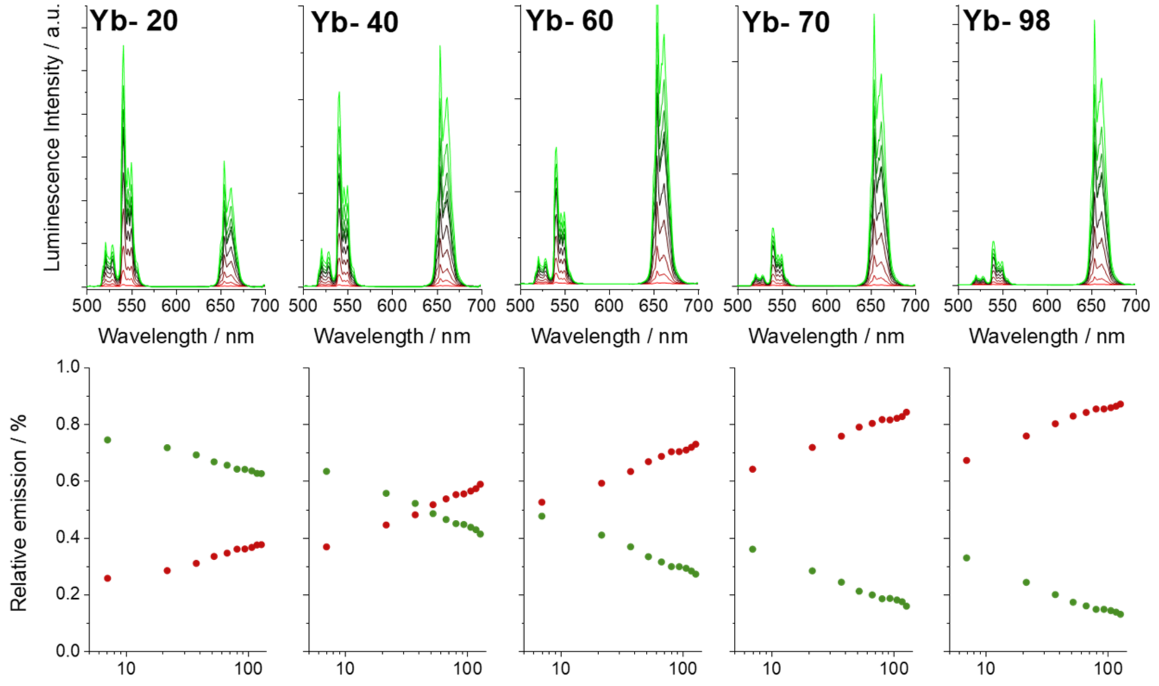

Figure S5: a) Excitation power dependency of the emission spectra of the samples from the Yb-dopant series. The color code represents the increasing excitation powers (red → green); b) Relative spectral contribution of the emission at 540 nm (green dots) and at 654 nm (red dots) to overall UCL as function of the excitation power density.

9. Synthesis of UCNPs starting from low-price Ln-precursors

a) As synthesized oleate-capped UCNPs

b) After ligand removal via acid treatment

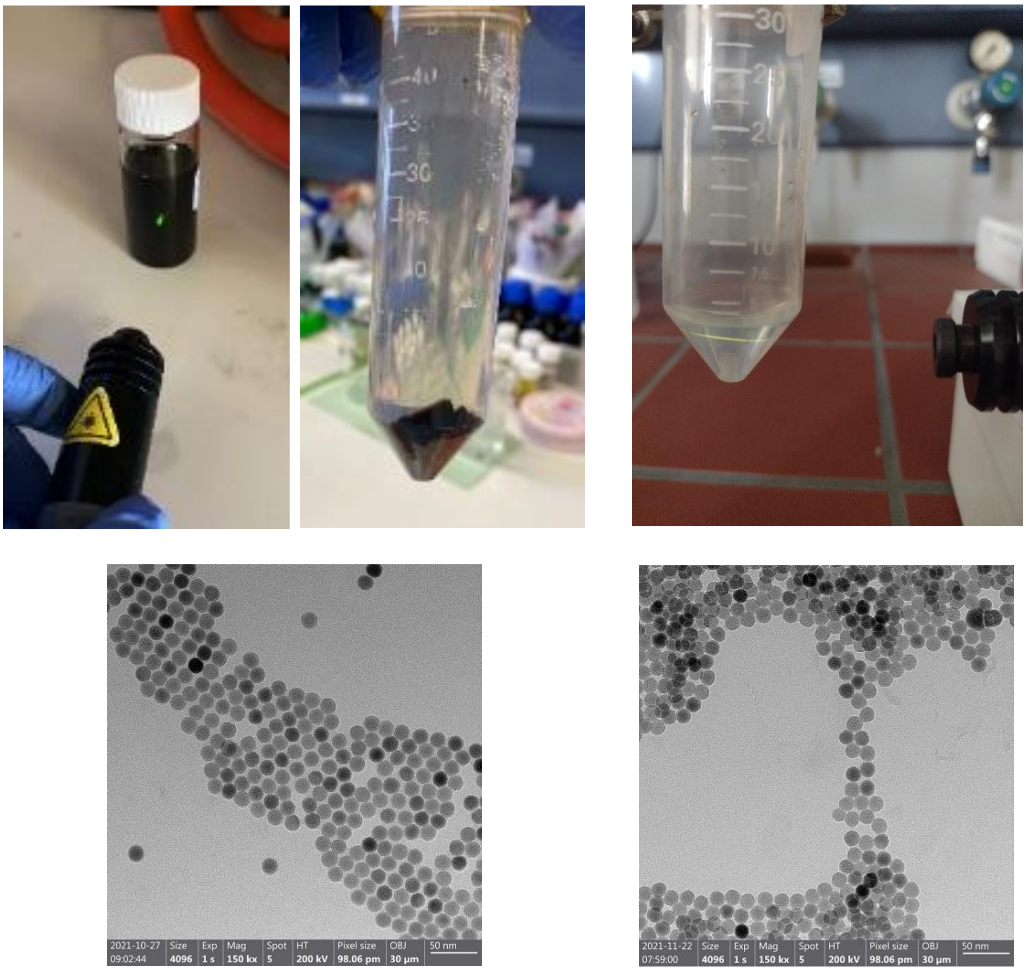

c)

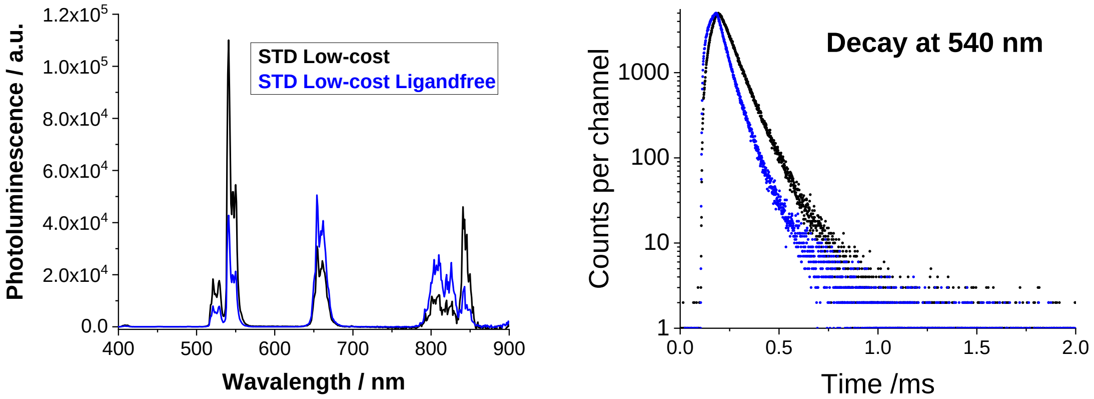

Figure S6: continued next page

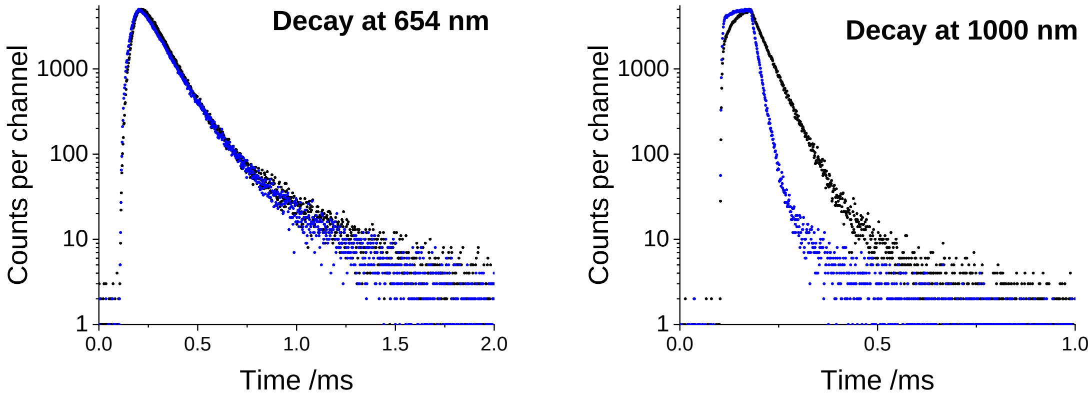

Figure S6: Photographs of a) as-synthesized UCNPs using low-price Ln-chlorides as precursors in dispersion in cyclohexane (left) and as solid (right) and b) after ligand removal via acid treatment and the corresponding TEM images (150kx magnification); c) Spectroscopic characterization of oleate-capped UCNPs prepared from low-cost precursors and after ligand removal.
